# A Cooperative Photoactive Class-I Hybrid Polyoxometalate With Benzothiadiazole–Imidazolium Cations

**DOI:** 10.3389/fchem.2020.612535

**Published:** 2021-01-14

**Authors:** Alexander J. Kibler, Virginia S. Souza, Jesum Alves Fernandes, William Lewis, Stephen P. Argent, Jairton Dupont, Graham N. Newton

**Affiliations:** ^1^GlaxoSmithKline Carbon Neutral Laboratories for Sustainable Chemistry, Department of Chemistry, University of Nottingham, Nottingham, United Kingdom; ^2^Laboratory of Molecular Catalysis, Institute of Chemistry, Universidade Federal do Rio Grande Do Sul, Porto Alegre, Brazil; ^3^Department of Chemistry, School of Chemistry, University of Nottingham, Nottingham, United Kingdom

**Keywords:** polyoxometalate, hybrid material, organic dye, electron transfer, class-I hybrid, photochemistry

## Abstract

An organic–inorganic hybrid species based on the Wells–Dawson polyoxotungstate [P_2_W_18_O_62_]^6−^ and novel fluorescent benzothiadiazole–imidazolium cations, [BTD-4,7-ImH]^2+^, has been synthesized. X-ray crystallographic analysis shows that the inorganic and organic components form a hydrogen-bonded superstructure and that the cations are revealed to be non-equivalent with varying degrees of rotation between the BTD and imidazolium rings due to competition between weak intra- and intermolecular interactions. The UV–vis diffuse reflectance spectra indicate that the hybrid has a band gap of 3.13 eV, while the solid-state fluorescence properties of the cation are quenched in the hybrid material, suggesting the existence of electron transfer between the inorganic and organic components. The highest occupied molecular orbital (HOMO) and lowest unoccupied molecular orbital (LUMO) energies of the polyoxometalate (POM) and BTD-4,7-ImH precursors, estimated through UV–vis absorption spectroscopy and cyclic voltammetry, indicate that electron transfer from the BTD cations to the POM may occur in the excited state.

## Introduction

Polyoxometalates (POMs) are discrete anionic metal oxide clusters, commonly formed from Group V and Group VI transition metals in their highest oxidation state. This vast family of compounds is reputed for their rich redox properties (Gumerova and Rompel, 2018) and photoactivity (Cameron et al., 2018) in conjunction with high thermal and oxidative stability (Varga et al., 1998; Lv et al., 2012). As such, POMs have demonstrated applicability in a wide number of research areas including redox and photoredox catalysis (Wang and Yang, 2015), optoelectronics (Chen et al., 2019), soft materials (Kastner et al., 2017), molecular magnetism (Baldoví et al., 2017), photochromic devices (Liu et al., 2006), hybrid nanomaterials (Jordan et al., 2019; Martin et al., 2020), and battery technologies (Huang et al., 2020).

Organic–inorganic hybrid POMs are an emerging family of molecules that involve the inclusion of organic moieties into the inorganic structure of the POM. Organic hybridization offers a near-limitless scope for the enhancement or modulation of the POMs properties through the intelligent design of the organic component. This is typically achieved in one of two ways: either via the exchange of alkali metal or proton countercations with organic countercations (Class I hybrid) or via the covalent grafting of organic fragments onto the POM (Class II hybrid) (Dolbecq et al., 2010; Kibler and Newton, 2018). Class I hybrids remain the most prolifically reported and studied due to their ease of synthesis through simple metathesis reactions and their compatibility with any anionic POM structure. Generally, the physical properties of the POMs are dominated and controlled by the cation, and thus, Class I hybridization is a highly attractive route toward POM-based functional materials such as POM ionic liquids (POM-ILs) (Kibler et al., 2019), POM charge transfer salts (Xu et al., 2010), and POM-decorated polymers (Herrmann et al., 2015).

A lesser explored avenue with Class I hybrids is the photosensitization of POMs using cationic organic or organometallic chromophores. While POMs possesses inherent photoactivity through excitation of the O → M ligand-to-metal charge transfer (LMCT) excitation, this is usually limited to the UV region with only marginal tailing into the visible region (Cameron et al., 2018). Cationic chromophores may not only serve to increase the visible light absorption profile of the material through its own excitations but may also allow for intermolecular charge transfer, which can give rise to new optical properties (Chong et al., 2017) or bolster photocatalytic performance (Zhang et al., 2015). Consequently, such materials have shown immense promise as artificial photosynthesis systems for both water oxidation (Bonchio et al., 2019) and carbon dioxide reduction (Ettedgui et al., 2011).

Amongst organic dye molecules, 2,1,3-benzothiadiazoles (BTDs) have recently burgeoned as a structural motif in a host of optical applications. Derivatives of BTD have been widely used as components in organic light-emitting diodes (OLEDs) (Fell et al., 2019) and organic solar cells (OSCs) (Yuan et al., 2019) as well as in bioimaging (Neto et al., 2015) and fluorescence sensing (Zhang et al., 2017). Recently, cationic BTD derivatives flanked by imidazolium moieties have shown impressive optical properties and were used as fluorescent lysosome-staining agents (Souza et al., 2020). The use of imidazole flanking groups on the photoactive core allowed for the formation of cationic derivatives without compromising the photoactivity of the system; such a motif would be highly intriguing as components of Class I hybrid POMs to form photoactive materials. Herein, we have synthesized a novel Class I hybrid material based on the combination of a Wells–Dawson phosphotungstate anion and an imidazolium-BTD di-cation [BTD-4,7-ImH]^2+^ and demonstrate the intimate electronic coupling exhibited between the two photoactive components.

## Materials and Methods

All reagents and solvents were purchased in high-purity grade from commercial sources and used without further purification. The preparation and characterization of K_6_P_2_W_18_O_62_ and [BTD-4,7-ImH]Cl_2_ were conducted according to previous reports (Contant et al., 1990; Souza et al., 2020). Nuclear magnetic resonance (NMR) spectroscopy was performed on a Bruker AV400 spectrometer at 298 K. Attenuated total reflectance–Fourier-transform infra-red (ATR-FTIR) spectroscopy was recorded on a Bruker Tensor 27 spectrometer equipped with a Pike GladiATR module. UV–vis spectroscopy was performed on an Agilent Cary 5000 UV–vis NIR Absorption spectrometer using a DRA-900 InGaAs integrating sphere. Fluorescence measurements were recorded on an Edinburgh Instruments FLS980 Photoluminescence spectrometer. Raman spectra were obtained using a HORIBA LabRAM HR Raman microscope, equipped with 532- and 785-nm lasers. CHN Elemental Microanalysis was performed on an Exeter analytical Ce-440 Elemental Analyzer by the UoN School of Chemistry elemental microanalysis services. Thermogravimetric analysis (TGA) and differential scanning calorimetry (DSC) were performed on a TGA Discovery Instrument using high-temperature platinum pans and sealed aluminum pans, respectively.

### X-Ray Crystallography

Single crystals were selected and mounted using Fomblin® (YR-1800 perfluoropolyether oil) on a polymer-tipped MiTeGen MicroMountTM and cooled rapidly to 120 K in a stream of cold N_2_ using an Oxford Cryosystems open-flow cryostat (Cosier and Glazer, 1986). Single-crystal X-ray diffraction (XRD) measurements were collected on an XtaLAB PRO MM007 (PILATUS3 R 200K Hybrid Pixel Array Detector, mirror-monochromated Cu-Kα radiation source; λ = 1.54184 Å, ω scans). Cell parameters were refined from the observed positions of all strong reflections in each data set, and absorption corrections were applied using a Gaussian numerical method with beam profile correction (CrysAlisPro). The structure was solved with Olex^2^ (Dolomanov et al., 2009) by dual-space iterative methods (SHELXT) (Sheldrick, 2015a) and all non-hydrogen atoms refined by full-matrix least-squares on all unique F2 values with anisotropic displacement parameters (SHELXL) (Sheldrick, 2015b). Hydrogen atoms were refined with constrained geometries and riding thermal parameters. Disordered solvent and counterion molecules within voids had their electronic contribution to the structure factor ascertained using the PLATON SQUEEZE procedure (Spek, 2015). The structure was checked with checkCIF. CCDC-2034694 contains the supplementary data for this compound. These data can be obtained free of charge from the Cambridge Crystallographic Data Centre via www.ccdc.cam.ac.uk/data_request/cif.

### Cyclic Voltammetry

Cyclic voltammetry was performed using a CHI600E workstation. A three-electrode setup was employed with a glassy carbon working electrode (*d* = 3 mm), platinum wire counter electrode, and a Ag wire pseudo reference electrode with the addition of ferrocene as an internal reference. All measurements were performed in anhydrous acetonitrile (10 ml) for solid-state measurements and anhydrous dimethylformamide (DMF) (10 ml) for solution-state measurements with 0.1 M of TBAPF_6_ as the supporting electrolyte.

The material was analyzed in the solid state by dropcasting the analyte in a carbon matrix onto the glassy carbon working electrode. For K_6_P_2_W_18_O_62_ and **1**, 5 mg of analyte is combined with 5 mg of Vulcan XC72 carbon and to this was added 20 μl of 10 wt% of poly(tetrafluoroethylene) (PTFE) binder in water and 200 μl of ethanol. The slurry is sonicated for 15 min, and then 5.5 μl of the suspension is immediately dropcast onto the center of the 3-mm glassy carbon and allowed to dry for 1 h. For [BTD-4,7-ImH]Cl_2_, 0.5 mg of analyte is combined with 5 mg of Vulcan XC72 carbon, and to this is added 10 μl of 10 wt% of PTFE binder in water and 100 μl of ethanol and prepared as above.

### Synthesis of 1

[BTD-4,7-ImH]Cl_2_ (0.44 g, 1.29 mmol) was dissolved in deionized water (15 ml). This was added dropwise to a stirring solution of K_6_P_2_W_18_O_62_ (2.09 g, 0.430 mmol) in deionized water (15 ml), which caused the immediate formation of a fine yellow precipitate. The reaction mixture was stirred overnight, and the fine yellow precipitate was collected by Buchner filtration. The precipitate was washed with deionized water and ethyl acetate to yield **1** as a yellow powder (0.737 g, 22%).To prepare single crystals, 60 mg of K_6_P_2_W_18_O_62_ is dissolved in 5 ml of DMF in a test tube; layered carefully onto this is 12.6 mg of [BTD-4,7-ImH]Cl_2_ in 5 ml of methanol; the tube is carefully sealed and left undisturbed for 1 week, precipitating **1** as yellow needles. ^1^H NMR (DMSO-*d*_6_, 400 MHz, ppm): δ = 9.15 (CH_Im_, s, 2H), 8.22 (CH_Im_-H, s, 4H), 7.57 (CH_Ar_, s, 2H). ^31^P NMR (DMSO-*d*_6_, 202 MHz, ppm): δ = −13.08. ATR-IR (cm^−1^): 3,133 (CH stretch aliphatic, w), 1,540 (C=N stretch aromatic, w) 1,370 (C=C stretch aromatic, w), 1,085 (P–O stretch, s), 954 (W=Od stretch, s), 901 (W–Ob–W, s), 730 (W–Oc–W). Anal. Calcd. for C_36_H_30_N_18_S_3_P_2_W_18_O_62_: C, 8.357; H, 0.584; N, 4.873. Found: C, 8.23; H, 0.56; N, 4.47.

## Results and Discussion

### Synthesis and Characterization

**Figure 1:**
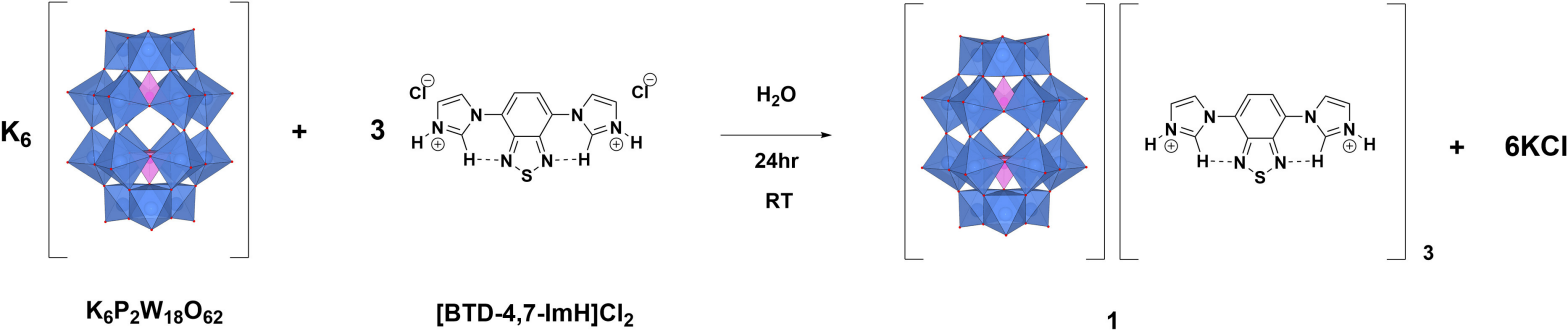
Synthetic scheme for the synthesis of **1**.

**1** is synthesized via salt metathesis (Figure 1). K_6_P_2_W_18_O_62_ and [BTD-4,7-ImH]Cl_2_ are separately dissolved in water in 1:3 molar ratios. The solution of [BTD-4,7-ImH]Cl_2_ is then added dropwise to the solution of K_6_P_2_W_18_O_62_, causing the immediate precipitation of [P_2_W_18_O_62_][BTD-4,7-ImH]_3_ (**1**) as a pale yellow solid. The integrity of the separate ions in **1** was confirmed by ^1^H and ^13^C NMR spectroscopies for the BTD-4,7-ImH cations and ^31^P for the P_2_W_18_O_62_ anions, and the expected stoichiometry and purity of the sample were confirmed with CHN microanalysis.

Initially, the photoabsorption profiles of K_6_P_2_W_18_O_62_, [BTD-4,7-ImH]Cl_2_, and **1** were evaluated with UV–vis absorption spectroscopy (Supplementary Figure 1). We can see that the POM exhibits minimal absorption in the visible region, whereas [BTD-4,7-ImH]Cl_2_ features a lower-energy absorption feature centered at 373 nm with appreciable tailing into the visible region; the hybrid **1** also exhibits this lower-energy absorption and therefore also absorbs in the high-energy visible region. We additionally screened hybrids of K_6_P_2_W_18_O_62_ formed with N-methylated and N-acetylated analogs of [BTD-4,7,-ImH]Cl_2_ for suitability as sensitizers. These were synthesized using the same methodology as above, giving **2** and **3** (see Supplementary Material for synthesis). We evaluated the absorptive properties of **1**, **2**, and **3** using solution UV–vis spectroscopy (Supplementary Figure 2); interestingly, **2** and **3** do not feature absorption bands that tail into the visible, and **1** has stronger absorptions in the 300- to 330-nm region. This prompted the further study of the structural and electronic properties of **1**.

The structure of **1** was further probed by FTIR and Raman spectroscopies. The FTIR spectrum of **1** compared with the starting materials (Supplementary Figure 3) clearly shows characteristic peaks that correspond to the inorganic anion (P–O, W=O, and W–O–W vibrational modes at ~1,085, ~955, and ~730 cm^−1^, respectively) and the organic cation (C–H stretching modes at >3,000 cm^−1^, C=C stretch at ~1,540 cm^−1^, and C=N stretch at ~1,370 cm^−1^). Similarly, the Raman spectrum of **1** (Supplementary Figure 4) also demonstrates the presence of both inorganic and organic components with prominent peaks from the POM (P–O stretch at ~995 cm^−1^ and W=O stretch at 975 cm^−1^) and the organic cation (BTD ring stretch at ~1,565 cm^−1^ and BTD C–H wag at ~1,345 cm^−1^).

Thermophysical characterization of **1** revealed that the material is stable up to 400°C at which point degradation of the organic cation is observed according to TGA (Ar atmosphere, Supplementary Figure 5). DSC also showed that the hybrid material undergoes no phase changes between 40 and 400°C despite containing imidazolium residues, which are common components of ILs, likely due to the highly symmetrical and sterically compact nature of the [BTD-4,7-ImH]^2+^ cations (see Supplementary Figure 6).

### Crystal Structure of 1

**Figure 2:**
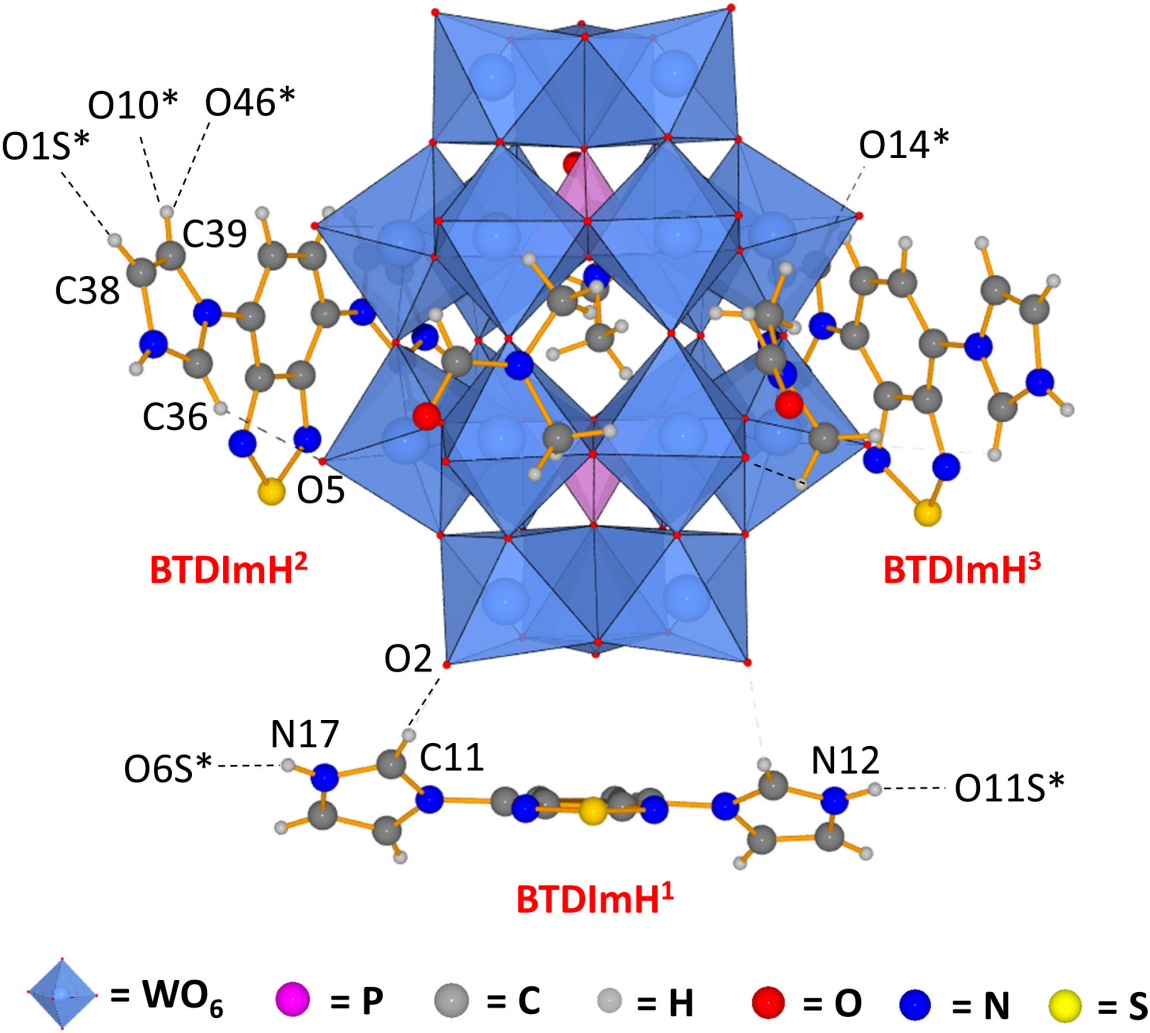
Asymmetric unit of **1** demonstrating that the three [BTD-4,7-ImH]^2+^ cations reside in distinct environments featuring different hydrogen bond contacts and different ring torsions. Dotted lines indicate hydrogen bonding; dotted lines to atom labels indicate hydrogen bonds with molecules beyond the asymmetric unit.

Single crystals of **1** were obtained by layering a solution of [BTD-4,7-ImH]Cl_2_ in methanol onto a solution of K_6_P_2_W_18_O_62_ in DMF, yielding yellow needles after 1 week. **1 c**rystallizes in a triclinic crystal system corresponding to the *P*$\bar{1}$ space group. Collection and refinement details and bond length, bond angle, hydrogen bond, and torsion angle tables can be found in the supporting information (Supplementary Tables 1–5). The asymmetric unit of **1** (Figure 2) consists of one Wells–Dawson polyoxotungstate anion [P_2_W_18_O_62_]^6−^, three [BTD-4,7-ImH]^2+^ cations, and three DMF solvent molecules that can be sensibly modeled. The residual electron density that cannot be assigned is calculated as 8 DMF molecules per asymmetric unit using PLATON SQUEEZE.

The three [BTD-4,7-ImH]^2+^ cations of the asymmetric unit reside in three locations: one is located close to the cap region of the POM (BTDImH^1^), whereas the other two reside closer to the belt regions (BTDImH^2^ and BTDImH^3^). No π-π stacking between the aromatic cations is observed. However, the cations make several hydrogen bonding contacts through the aromatic protons with oxygen atoms on the solvent or the POM (see Supplementary Table 4). The shortest hydrogen-bonded interactions are the imidazolium N–H protons of BTDImH^1^ with the amidic oxygen of the solvent [N12-O11S; 2.54(5) Å, N17-O6S; 2.676 Å] and the acidic C–H proton of the imidazolium ring with the terminal oxo group of the W-O on the POM cap [C11-O2; 3.06(4) Å]. BTDImH^2^ and BTDImH^3^ also feature short-range contacts between their non-acidic C–H protons and oxygen atoms of the solvent and POM. However, regardless of whether their bond lengths exceed 3.1 Å or their bond angles deviate from linearity by >20°, these are likely to be dictated largely by crystal packing considerations. Apart from their position, the three cations also differ from each other with respect to the degree of ring torsion between the imidazolium rings and the BTD aromatic core. All of the [BTD-4,7-ImH]^2+^ cations show significant deviation from planarity due to the presence of strong hydrogen bond acceptor groups on both the POM (terminal oxo) and the solvent (amide oxygen); in general, the degree of ring twisting shows a positive correlation with the number of hydrogen bonds that the imidazolium ring forms (Supplementary Tables 4, 5). For comparison, the crystal structure of [BTD-4,7-ImH]Cl_2_, which has been published previously (Souza et al., 2020), shows each imidazolium ring making multiple hydrogen bonding contacts with proximal chloride anions, giving large ring torsion angles (Supplementary Figure 7). The presence of hydrogen bonding contacts between the POM and the organic cation that alters its structure demonstrates that the two components interact strongly in the solid state.

### Electronic Characterization of 1

**Figure 3:**
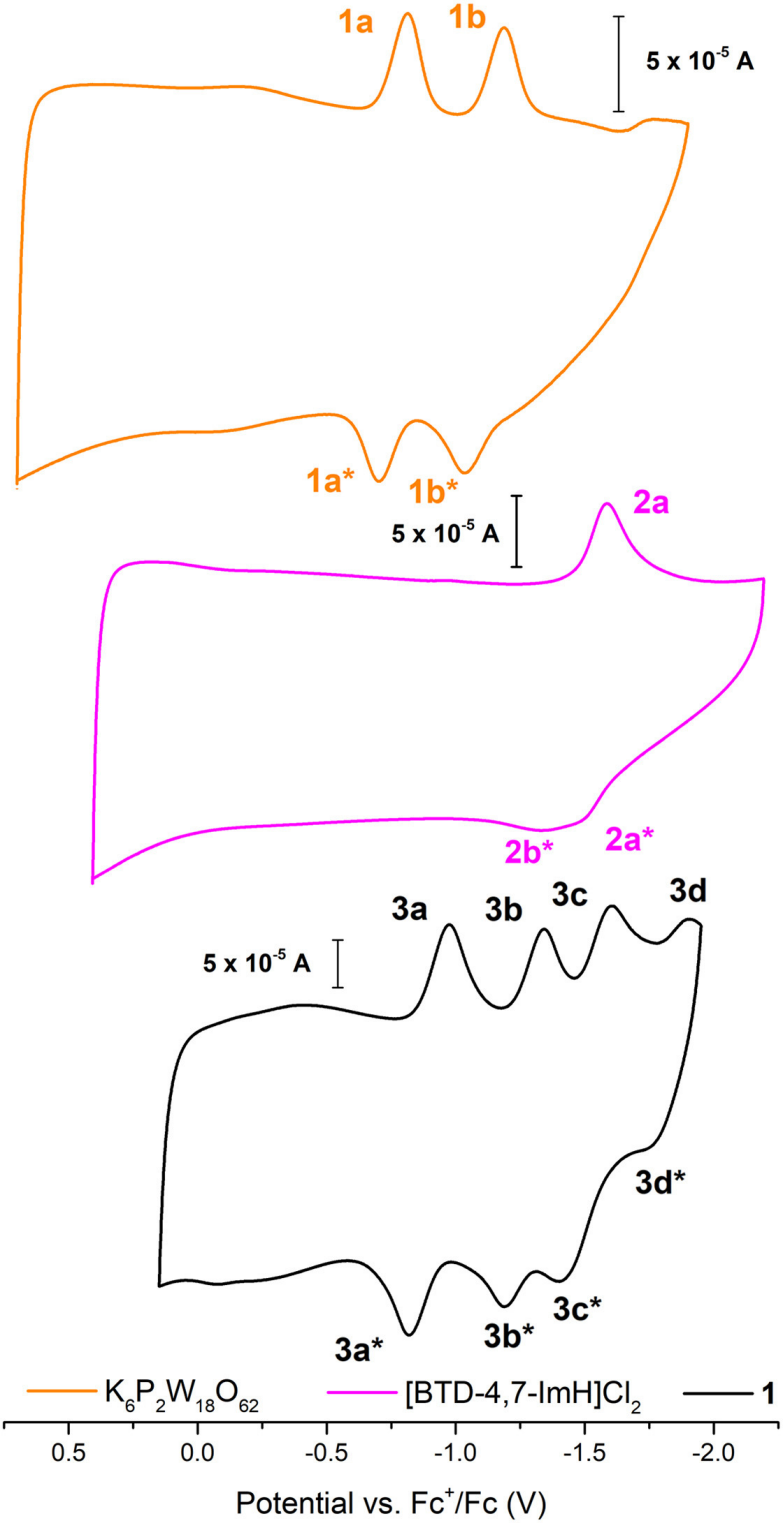
Solid-state cyclic voltammetry of 1 K_6_P_2_W_18_O_62_ (orange), [BTD-4,7-ImH]Cl_2_ (pink), and **1** (black) performed in anhydrous acetonitrile (10 ml) with 0.1 M of TBAPF_6_ as supporting electrolyte and reported vs. Fc^+^/Fc as an internal reference.

The electrochemical nature of **1** was probed using cyclic voltammetry. Given the electrical non-conductivity of **1** and its starting materials, it was necessary to prepare the solids as a carbon ink, which was immobilized to the surface of the glassy carbon. Figure 3 shows the cyclic voltammetry of K_6_P_2_W_18_O_62_, [BTD-4,7-ImH]Cl_2_, and **1**; redox events have been labeled; and their potentials are given in Table 1. Firstly, in each voltammogram, it is immediately clear that there is a large contribution to the current due to the capacitive charging of the carbon matrix. This somewhat obfuscates the redox signals from the active species and makes measurements of the peak area difficult, so we have focused solely on the values of peak potential.

**Table 1:** Redox potentials measured for K_6_P_2_W_18_O_62_, [BTD-4,7-ImH]Cl_2_, and **1** according to the labeling scheme in Figure 3.

**K**_****6****_**P**_****2****_**W**_****18****_**O**_****62****_		**[BTD-4,7-ImH]Cl**_****2****_		**1**			
**Peak**	**Potential (V)**	**Peak**	**Potential (V)**	**Peak**	**Potential (V)**	**Peak**	**Potential (V)**
1a	−0.814	2a	−1.586	3a	−0.975	3c	−1.607
1a^*^	−0.704	2a^*^	−1.498	3a^*^	−0.819	3c^*^	−1.400
1b	−1.188	2b^*^	−1.330	3b	−1.344	3d	−1.900
1b^*^	−1.035			3b^*^	−1.189	3d^*^	−1.755

*Reported as the peak maximum (Ep) in volts (V) referenced to Fc^+^/Fc as the internal standard*.

* *Return oxidation associated with a given process*.

K_6_P_2_W_18_O_62_ shows two reductions with two associated oxidation peaks within the potential window, in accordance with solution state measurements of K_6_P_2_W_18_O_62_ in aprotic media; these correspond to two reversible one-electron reductions (Supplementary Figure 8). The electrochemistry of [BTD-4,7-ImH]Cl_2_ shows one reduction peak with a corresponding oxidation peak; the peak shape of the oxidation is suggestive of two overlapping one-electron processes, indicating that the reduction may be multielectron. The electrochemistry of the hybrid **1** shows four reduction peaks with associated oxidation peaks, the first two peaks (**3a**/**3b**) relate to POM-based electrochemical processes, and the third peak (**3c**) matches the organic cation reduction process seen in **3b**. The final peak is likely to be the third reduction peak of the POM as seen in Supplementary Figure 6. Its appearance in the voltammogram of **1** may be the result of superior charge compensation of the more reduced states due to intimate packing of the organic BTD cations compared with potassium cations. Apart from this additional peak, the profile of **1** represents an overlay of the component salts with a slight negative shift in peak potential for each process (see Table 1).

**Figure 4:**
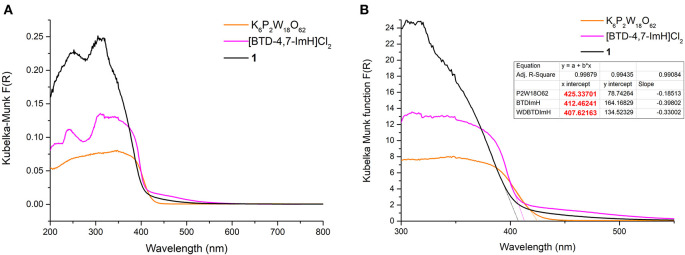
**(A)** Diffuse reflectance UV–vis spectra of K_6_P_2_W_18_O_62_ (orange), [BTD-4,7-ImH]Cl_2_ (pink), and **1** (black). **(B)** Magnification of the diffuse reflectance UV–vis spectra with extrapolation of the linear portion of the graph giving the x-intercept (see dotted lines and table inset).

The photoactivity of **1** and its starting materials were investigated via diffuse reflectance spectroscopy. The diffuse reflectance spectrum of K_6_P_2_W_18_O_62_ exhibits multiple broad overlapping absorptions between 200 and 430 nm corresponding to various LMCT transitions within the structure in accordance with the literature (Ross-Medgaarden and Wachs, 2007). The organic cation [BTD-4,7-ImH]Cl_2_ showed a typical profile of an aromatic framework with bands at 241, 310, and ~340 nm representing various π-π^*^ transitions; this correlates well with the absorption bands measured in the solution (Supplementary Figure 1). However, the tailing of the lower-energy absorption in the solution state is not reflected in the solid state. This may be due to the slight distortion of the BTD-4,7-ImH molecules in the crystalline salt (Supplementary Figure 7) (Souza et al., 2020). With this in mind, we note that solution-phase analysis alone in the preparation of hybrid materials can at times paint only half of the picture, particularly when targeting potential applications in heterogeneous photocatalysis. The diffuse reflectance spectrum of **1** shows two prominent bands at 252 and 305 nm. These roughly correlate to absorption bands observed for the two photoactive components, and thus, no new bands arise from the combination of the POM with the organic cations. Nonetheless, it can be seen from Figure 4A that the absorbance of **1** in the UV region is considerably higher than that of either of the starting materials due to the pairing of two photoactive components.

**Figure 5:**
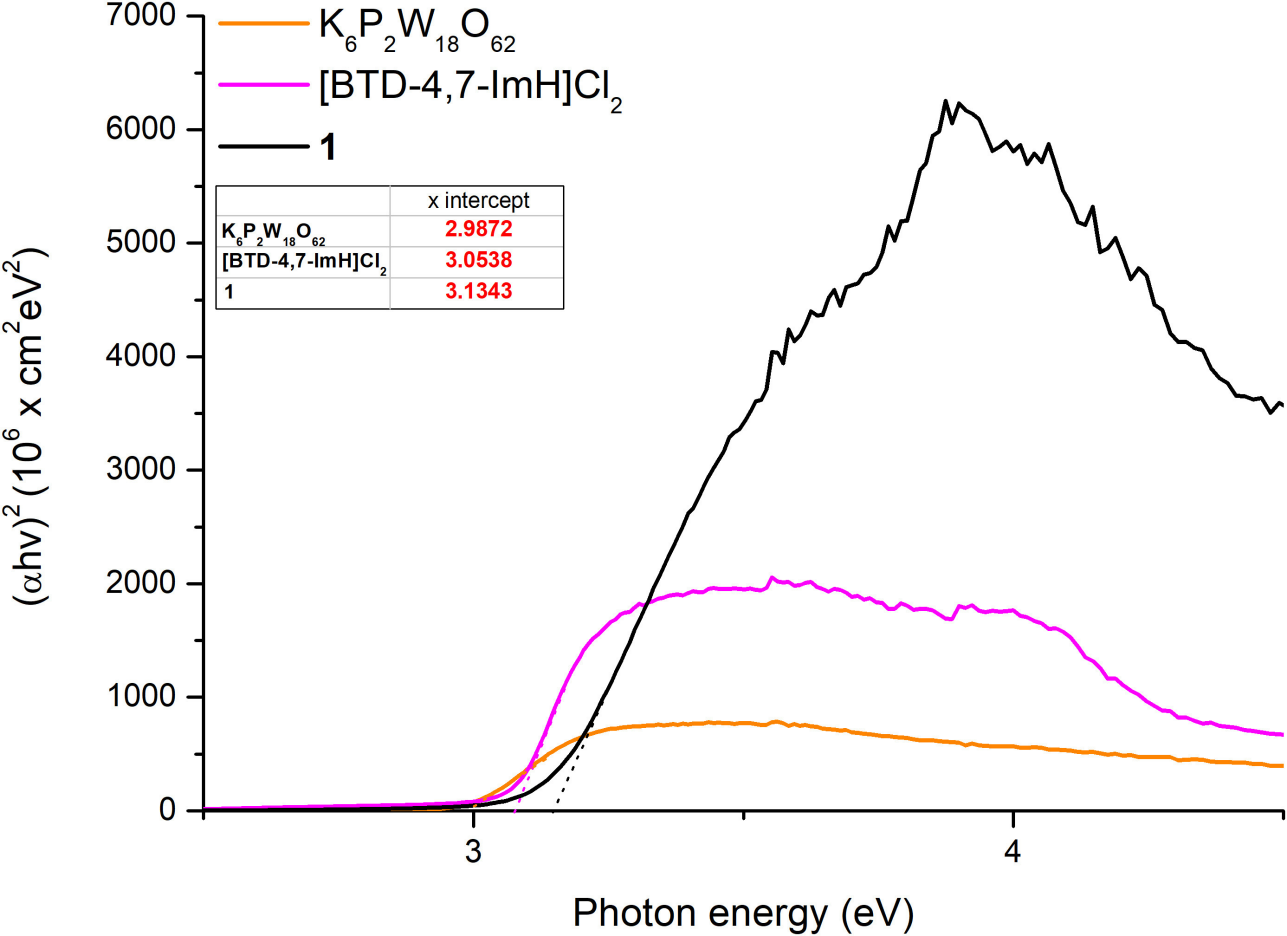
Tauc plots of K_6_P_2_W_18_O_62_ (orange), [BTD-4,7-ImH]Cl_2_ (pink), and **1** (black) with extrapolation of the linear portion of the graph giving the x-intercept (see dotted lines and table inset).

The absorption edges of **1** and the starting materials were also calculated using the Kubelka–Munk function (Mirabella, 1998) as shown in Figure 4B. Interestingly, it was observed that the absorption edge of **1** (408 nm) is slightly blue shifted as compared with K_6_P_2_W_18_O_62_ (425 nm) and [BTD-4,7-ImH]Cl_2_ (412 nm). This phenomenon was also observed in the calculated band gap from the Tauc plots (Viezbicke et al., 2015) (Figure 5), with **1** exhibiting the largest band gap of 3.13 eV, and K_6_P_2_W_18_O_62_ and [BTD-4,7-ImH]Cl_2_ showed smaller band gaps of 2.98 and 3.05 eV, respectively. The larger band gap and higher absorption of **1** mean that the hybrid material could be of interest as a UV photocatalyst.

**Figure 6:**
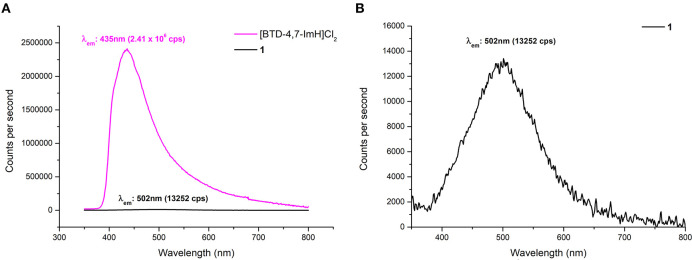
**(A)** Solid-state fluorescence emission spectra of [BTD-4,7-ImH]Cl_2_ (pink) and **1** (black) with excitation at 305 nm. **(B)** Magnification of the fluorescence emission spectrum of **1** showing the emission maximum.

Due to the reported fluorescent properties of [BTD-4,7-ImH]Cl_2_ in the solution (Souza et al., 2020), we were interested in investigating the fluorescence properties of the organic di-cation and the POM hybrid material **1** in the solid state (Figure 6). K_6_P_2_W_18_O_62_ shows no fluorescence in the solid state, whereas [BTD-4,7-ImH]Cl_2_ shows a fluorescence peak at 435 nm with relative intensity of 2.41 × 10^6^ cps when excited at its absorption maxima (310 nm), giving a Stokes shift of 125 nm. In contrast, **1** demonstrates a much weaker fluorescence at 502 nm with a relative intensity of 1.33 × 10^4^ cps when excited at its absorption maxima (305 nm), resulting in a much larger Stokes shift of 197 nm. We also investigated the fluorescence behavior of [BTD-4,7-ImH]Cl_2_ and **1** at lower excitation wavelengths (300, 350, 400, and 450 nm) (Supplementary Figure 9). [BTD-4,7-ImH]Cl_2_ shows a decay in fluorescence intensity moving toward lower energy excitation wavelengths as expected for a simple one-component system. Interestingly, the fluorescence intensity and peak maximum of **1** show considerable fluctuation based on the irradiation wavelength, likely due to differences in excited state populations between the two components, which affect the degree and mechanism of quenching.

A reduction in fluorescence from **1** is to be expected purely based on the reduced molar equivalent of fluorescent species vs. [BTD-4,7-ImH]Cl_2_ (16 vs. 79 wt%). However, this does not justify the ~200-fold reduction of fluorescence intensity, which is likely the static quenching of the [BTD-4,7-ImH]^2+^ excited state by [P_2_W_18_O_62_]^6−^, as has been observed in other solid-state Class I POM hybrids (Menet et al., 2015). Thus, the absence of a charge transfer in the absorption profile but fluorescence quenching in the excited state suggests that excited-state electron transfer from [BTD-4,7-ImH]^2+^ to [P_2_W_18_O_62_]^6−^ is occurring. This observation is further supported by the new weak red-shifted emission, which has been similarly observed between adducts of [Ru(bpy)_3_]^2+^ with Wells–Dawson POMs and originates from emission of the adduct species following excited electron charge transfer (Keyes et al., 2003; Seery et al., 2004). The observed Stokes shift from a POM-based adduct with a luminescent cation is the largest reported to date, demonstrating the strong electronic interaction between the ions in the excited state. Overall, the wavelength-dependent behavior of **1** coupled with the large Stokes shift suggests a more intimate photophysical relationship beyond that expected by two components that independently compete for absorption of light at similar wavelengths.

To explore the feasibility of excited electron transfer between [BTD-4,7-ImH]^2+^ and [P_2_W_18_O_62_]^6−^ and the electronic nature of **1**, the frontier orbital energies were estimated from the first reduction potential and the absorption edge. The lowest unoccupied molecular orbital (LUMO) energy is correlated to the first reduction potential, and the highest occupied molecular orbital (HOMO)–LUMO gap is calculated from the lowest energy electronic transition using Equations (1) and (2), respectively (Riaño et al., 2014).

(1) $$\begin{array}{l} E_{LUMO}\left(eV\right)\;=\;-\left(E_{red}\;+\;4.80\right) \end{array}$$

(2) $$\begin{array}{l} E_{g}\left(eV\right)\;=\;\frac{1,240}{\lambda_{onset}} \end{array}$$

**Figure 7:**
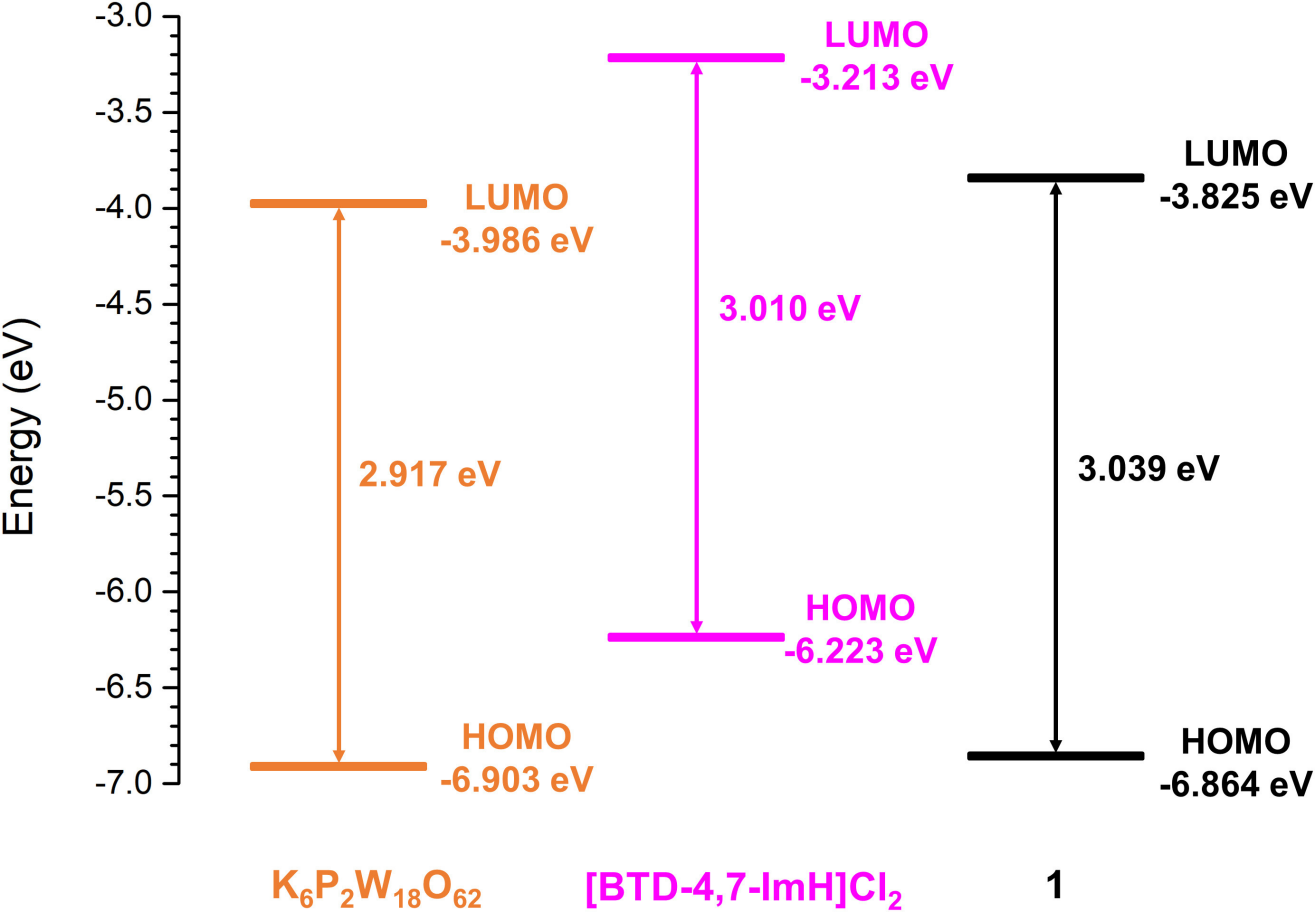
Derived frontier orbital energies for K_6_P_2_W_18_O_62_, [BTD-4,7-ImH]Cl_2_, and **1**.

The frontier orbital energies of K_6_P_2_W_18_O_62_, [BTD-4,7-ImH]Cl_2_, and **1** are summarized in Figure 7. Compared with K_6_P_2_W_18_O_62_, the HOMO and LUMO of **1** have become slightly stabilized and destabilized, respectively. The minimal changes to the HOMO–LUMO energies of **1** compared with K_6_P_2_W_18_O_62_ and the lack of observable charge transfer phenomena suggest that the components are electronically isolated in the ground state with no orbital mixing; however, the drastic change in fluorescence behavior is indicative of electron transfer in the excited state.

**Figure 8:**
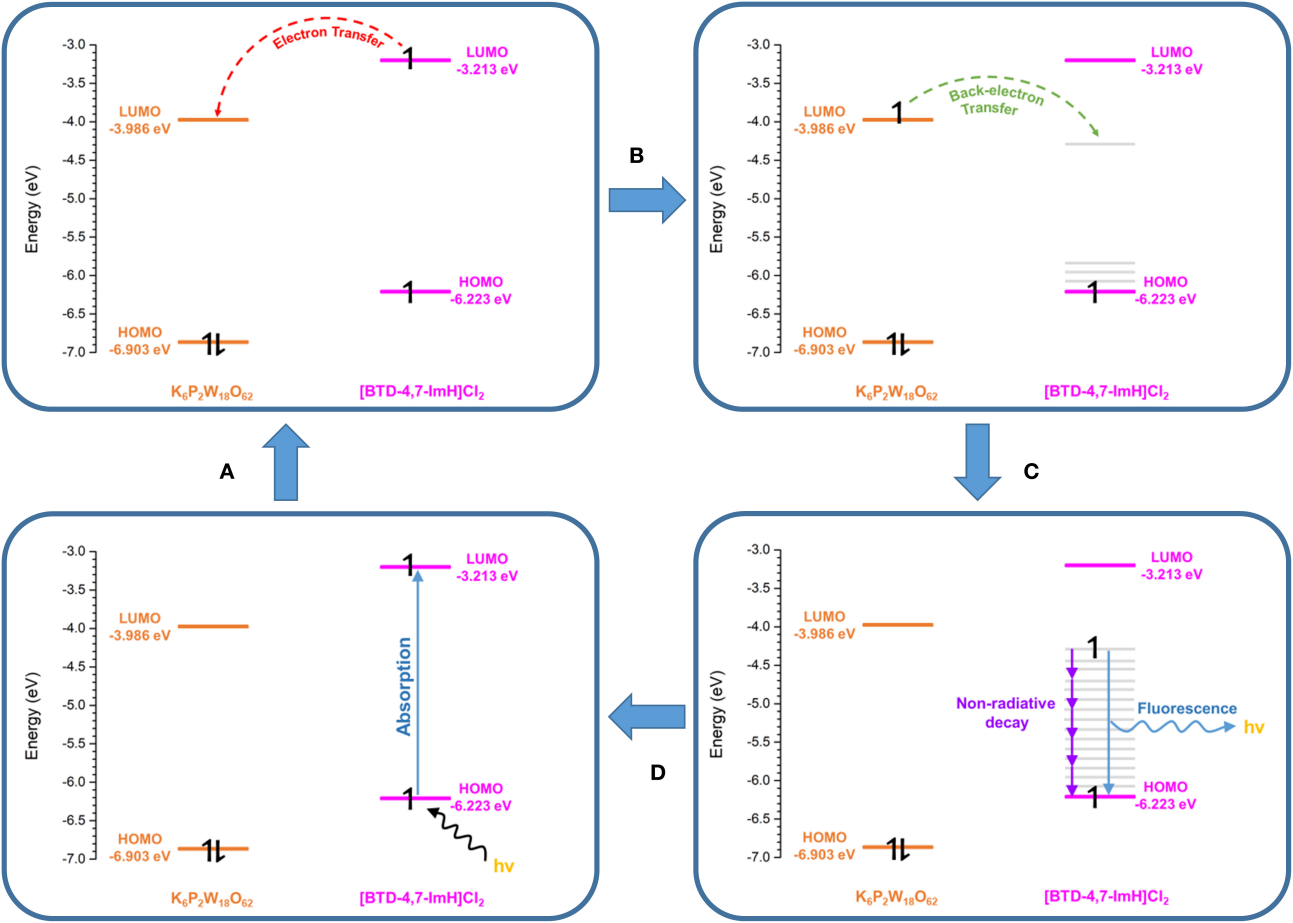
Proposed photoexcitation mechanism of **1**. **(A)** Photoexcitation of the [BTD-4,7-ImH]^2+^ cation. **(B)** Charge transfer from the [BTD-4,7-ImH]^2+^ cation to the polyoxometalate (POM). **(C)** Back electron transfer from the POM to a lower-energy meta-stable state of the [BTD-4,7-ImH]^2+^ cation. **(D)** Relaxation of the excited state via fluorescence (minor) and non-radiative decay (major).

To explore this, we considered the photoexcitation behavior of **1** based on the frontier orbital energies of the component salts. In the ground state, the HOMO of [BTD-4,7-ImH]Cl_2_ is lower in energy than the LUMO of K_6_P_2_W_18_O_62_ and so is the HOMO of K_6_P_2_W_18_O_62_ relative to the LUMO of the organic component. Accordingly, there is a large energetic barrier for charge transfer in either direction in the ground state. However, once the organic cation undergoes photoexcitation, the electron is promoted to the LUMO, which lies above the LUMO of the POM (0.773 eV driving force), thus allowing for charge transfer in the excited state. We propose that the charge separated state eventually relaxes to the ground state through back-electron transfer to the HOMO of the organic cation via a low-energy meta-stable state, leading to the observation of a new fluorescence band with a large Stokes shift. The emission is heavily quenched, likely due to competing non-radiative relaxation mechanisms (Figure 8).

## Conclusions

In summary, we have synthesized a cooperative photoactive Class I POM hybrid using the Wells–Dawson POM anion [P_2_W_18_O_62_]^6−^ and a fluorescent BTD cationic dye [BTD-4,7-ImH]^2+^. The resultant hybrid, **1**, shows strong interaction of the inorganic and organic components via electrostatic association and hydrogen bonding contacts as seen by single-crystal XRD. The optical properties of **1** show absorption features of both components; however, the resultant material had a larger band gap than either of the starting materials. The fluorescence of the [BTD-4,7-ImH]^2+^ cations was heavily quenched in the solid state upon association with the POM; however, no charge transfer character is observed in the absorption spectrum. Furthermore, the weakened fluorescence of **1** has a very large Stokes shift as compared with that of [BTD-4,7-ImH]^2+^, indicating that electron transfer occurs from the dye molecule to the POM upon photoexcitation. The feasibility of such a process was confirmed by estimating the frontier orbitals of the two components and demonstrating that the LUMO of the [BTD-4,7-ImH]^2+^ cation was higher in energy than the LUMO of [P_2_W_18_O_62_]^6−^, thus providing a thermodynamic driving force for such an event to occur and validating the observed difference in fluorescence behavior. Given the high absorption intensity between 300 and 425 nm and large band gap of **1**, we are currently investigating the hybrid as a heterogeneous artificial photosynthesis catalyst.

## Data Availability Statement

The datasets presented in this study can be found in online repositories. The names of the repository/repositories and accession number(s) can be found in the article/Supplementary Material.

## Author Contributions

AK performed the experimental work and data interpretation and wrote the manuscript. VS aided in the preparation of the BTD materials. JF assisted in the solid-state photochemical measurements and data interpretation. WL and SA assisted in crystallography measurements and solving the structure. JD contributed to the development of the BTD systems and contributed to project discussions. GN devised the project and co-wrote the manuscript. All authors contributed to the article and approved the submitted version.

## Conflict of Interest

The authors declare that the research was conducted in the absence of any commercial or financial relationships that could be construed as a potential conflict of interest.
