# Supplementary material for: A Cooperative Photoactive Class-I Hybrid Polyoxometalate With Benzothiadiazole–Imidazolium Cations
Source: Front Chem. 2021 Jan 14;8:612535. doi: 10.3389/fchem.2020.612535 (PMC7841050; doi:10.3389/fchem.2020.612535)
Supplement: Supplementary file 1 [file Table_1.DOCX]

Supplementary Material

**Synthesis of 2**

[BTD-4,7-ImMe]I_2_ (0.60 g, 1.29 mmol) was dissolved in a mixture of deionised water (20 ml) and acetone (10 ml). This was added dropwise to a stirring solution of K_6_P_2_W_18_O_62_ (2.09 g, 0.430 mmol) in deionised water (10 ml) which caused the immediate formation of a creamy orange precipitate. The reaction mixture was stirred overnight, and the orange precipitate was collected by Buchner filtration. The precipitate was washed with deionised water and ethyl acetate to yield **2** as a pale orange powder (1.69 g, 71%). ^1^H NMR (DMSO-d_6_, 400MHz, ppm): δ = 10.06 (CH_Im_, m, 2H), 8.52 (CH_Im_, m, 2H), 8.44 (CH_Ar_, s, 2H), 8.08 (CH_Im_, s, 2H) 4.11 (CH_3_, s, 6H). ^31^P NMR (DMSO-d6, 202 MHz, ppm): δ = -13.15. ATR-IR (cm^-1^): 3105 (CH stretch aliphatic, w), 1582 (C=N stretch aromatic, w) 1349 (C=C stretch aromatic, w), 1088 (P-O stretch, s), 954 (W=Od stretch, s), 907 (W-Ob-W, s), 763 (W-Oc-W).

**Synthesis of 3**

[BTD-4,7-ImAc]Cl_2_ (0.59 g, 1.29 mmol) was dissolved in a mixture of deionised water (10 ml) and acetone (10 ml). This was added dropwise to a stirring solution of K_6_P_2_W_18_O_62_ (2.09 g, 0.430 mmol) in deionised water (15 ml) which caused the immediate formation of a pale yellow precipitate. The reaction mixture was stirred overnight, and the pale yellow precipitate was collected by Buchner filtration. The precipitate was washed with deionised water and ethyl acetate to yield **3** as a pale yellow powder (0.737 g, 22%). ^1^H NMR (DMSO-d_6_, 400MHz, ppm): δ = 10.15 (CH_Im_, m, 2H), 8.58 (CH_Im_, m, 2H), 8.51 (CH_Ar_, s, 2H), 8.13 (CH_Im_, s, 2H), 5.36 (CH_2_, s, 4H). ^31^P NMR (DMSO-d6, 202MHz, ppm): δ = -13.13. ATR-IR (cm^-1^): 3363 (O-H stretch, b), 1732 (C=O stretch, m), 1621 (O-H bend, m), 1549 (C=N stretch aromatic, w), 1085 (P-O stretch, s), 954 (W=Od stretch, s), 903 (W-Ob-W, s), 761 (W-Oc-W).

**

Figure S1.** Solution-state UV-vis spectra of K_6_P_2_W_18_O_62_ (orange), [BTD-4,7-ImH]Cl_2_ (pink) and **1** (black) as 1x10^-5^ M solutions in DMSO.

**Figure S2.** Solution-state UV-vis spectra of K_6_P_2_W_18_O_62_ (black), **1** (red), **2** (blue) and **3** (green) as 1x10^-5^ M solutions in DMSO.**

**


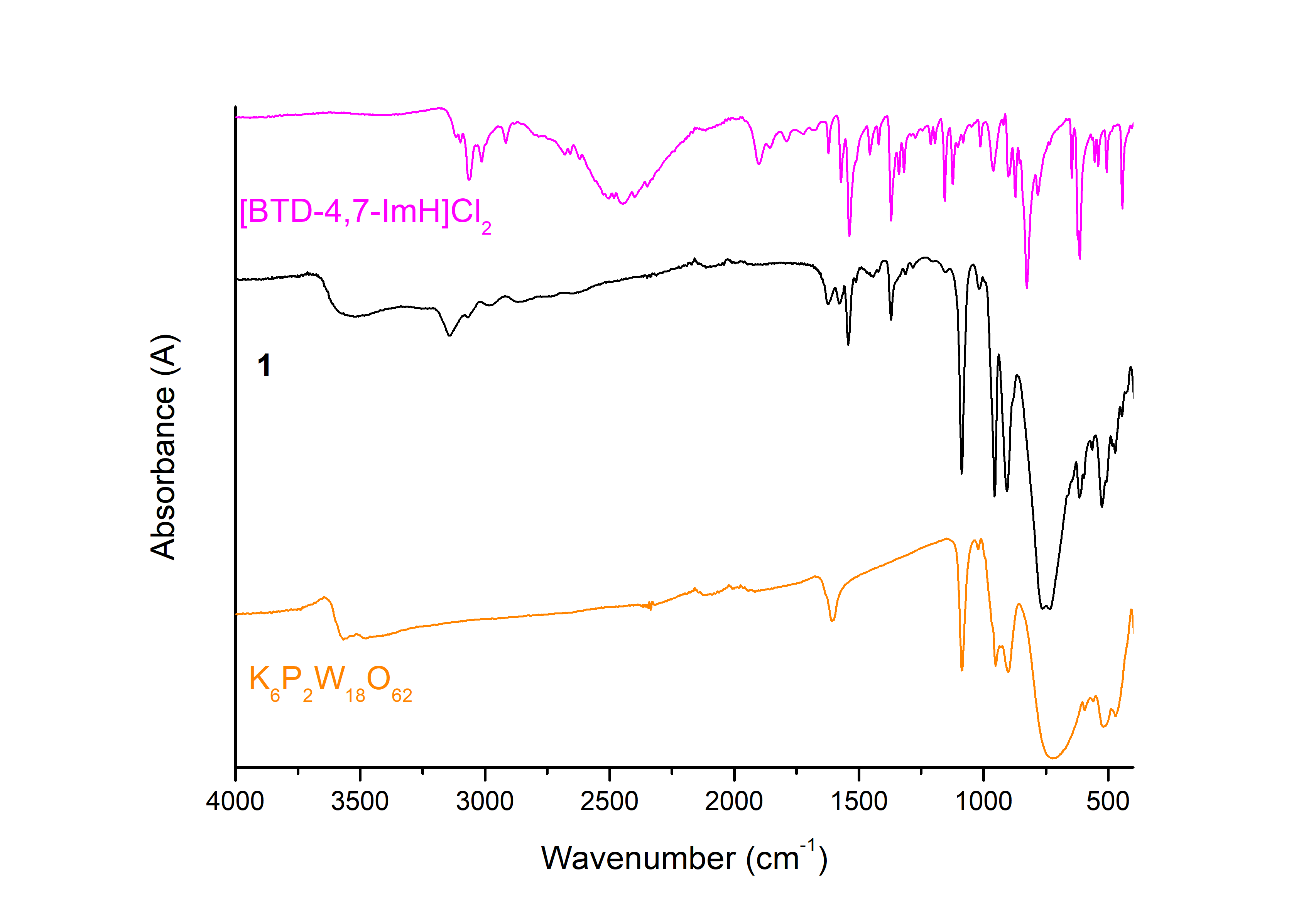
**Figure S3.** ATR-IR spectrum of K_6_P_2_W_18_O_62_ (orange), [BTD-4,7-ImH]Cl_2_ (pink) and **1** (black).


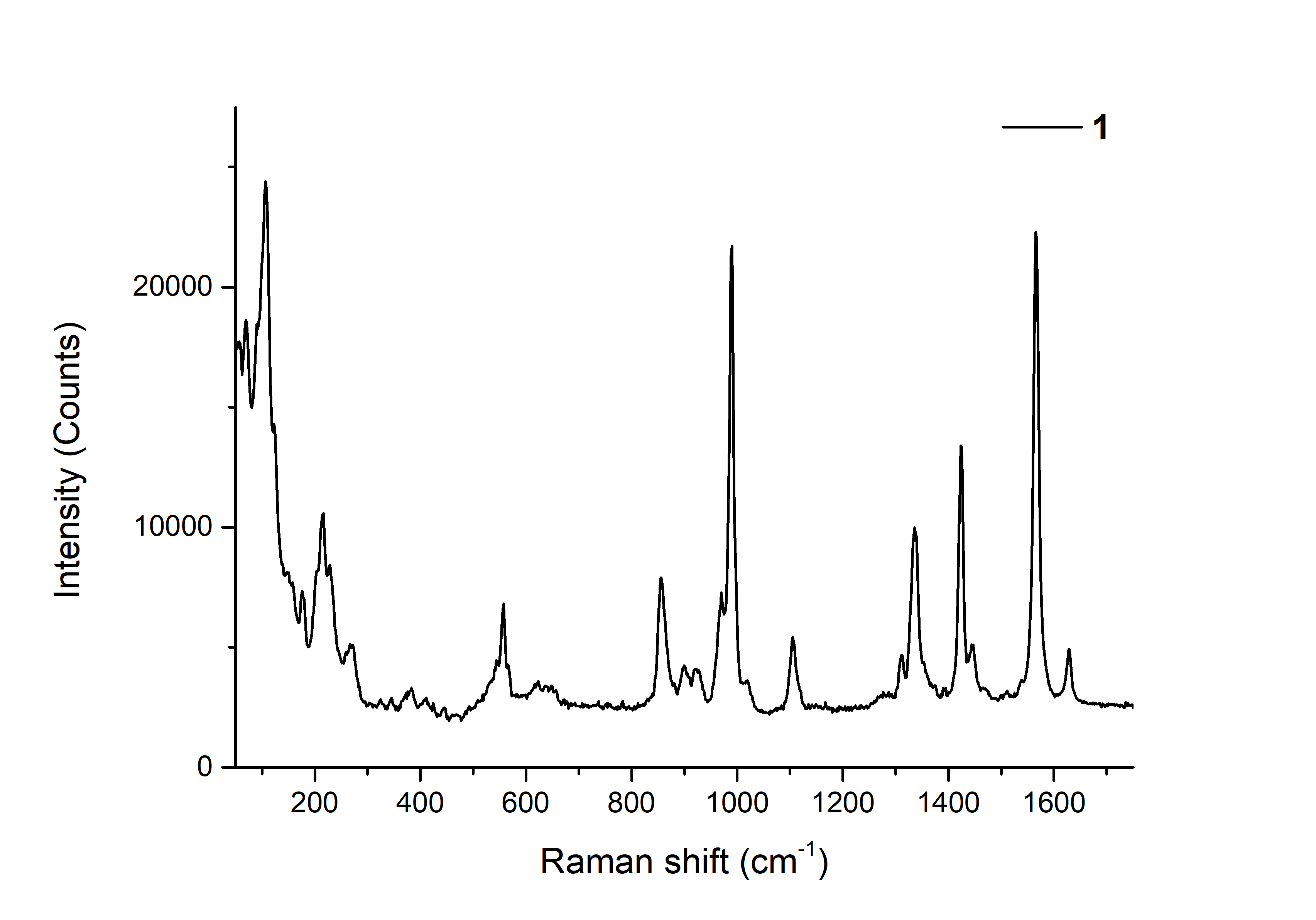
 **Figure S4.** Raman spectrum of **1** with excitation wavelength of 532 nm.


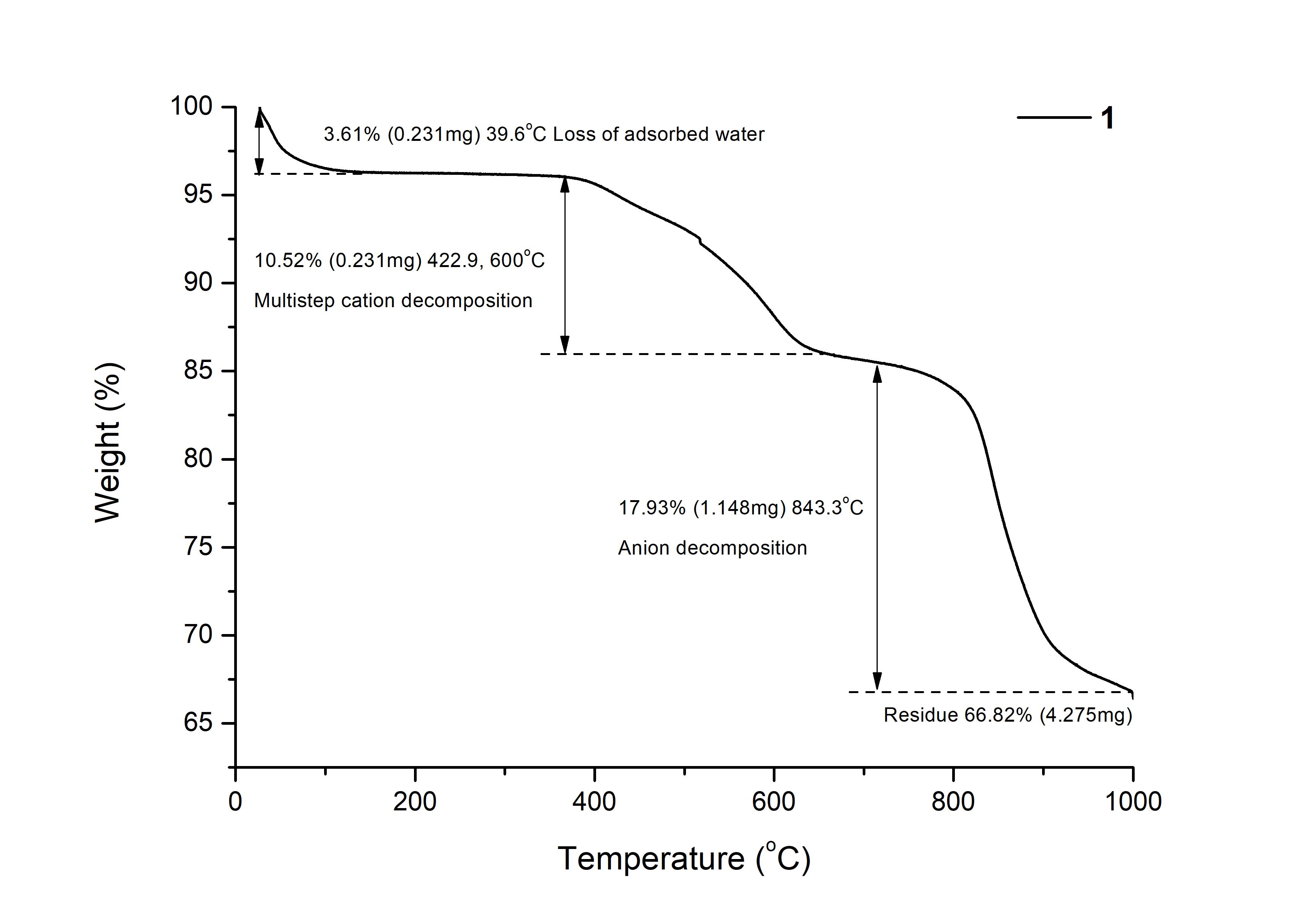
 **Figure S5**. TGA of **1** performed under an Argon atmosphere at a ramp rate of 10ºC/min from 25ºC to 1000ºC.


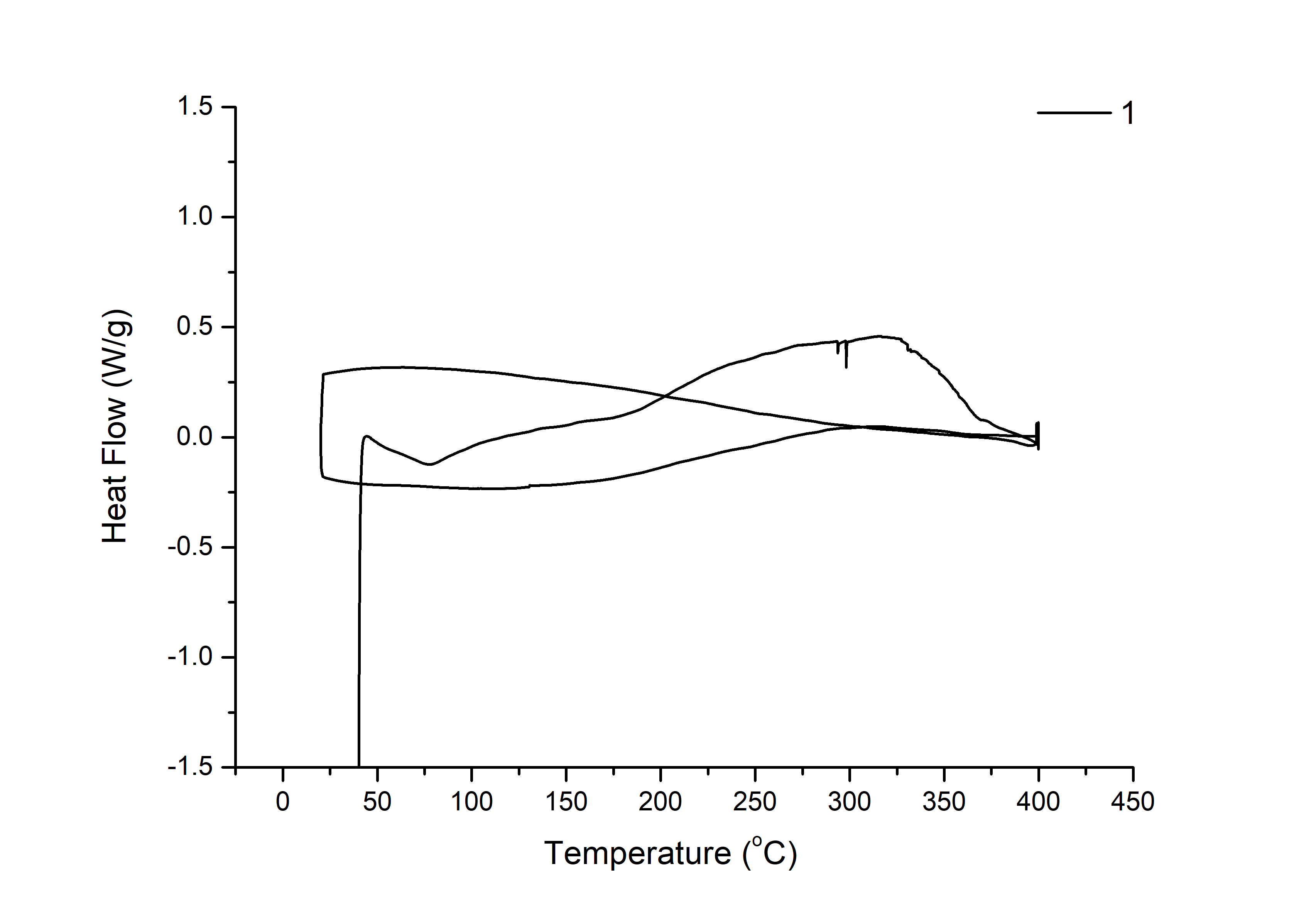
 **Figure S6**. DSC of **1** at a ramp rate of 10ºC/min from 25ºC to 400ºC with two heating ramps and one cooling ramp.


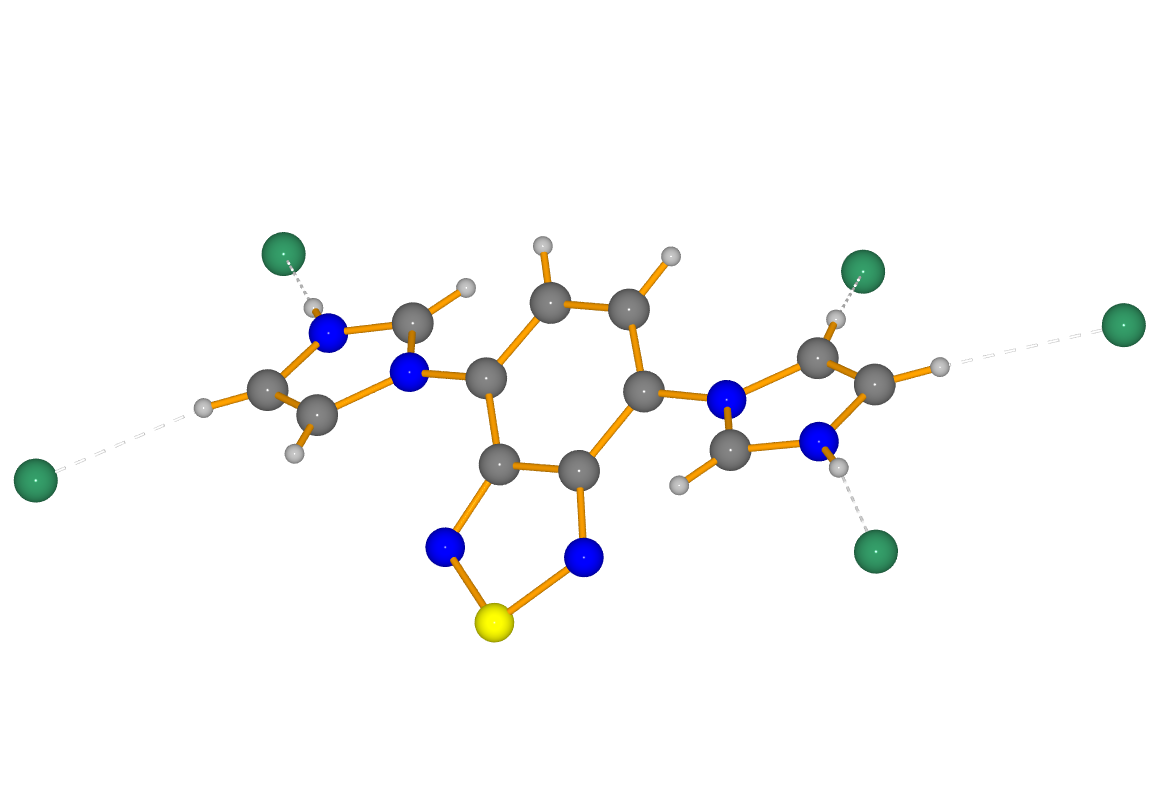


2.57 Å, 172.4º

2.17 Å, 174.9º

2.58 Å, 157.9º

2.62 Å, 176.7º

2.16 Å, 171.6º

**Figure S7.** Crystal structure of [BTD-4,7-ImH]Cl_2_ obtained from the CIF file as published by Souza et al. (Souza et al., 2020) CCDC entry MUVGES #1436542. Dotted line show hydrogen bonding contacts between imidazolium C-H and N-H bonds with D-H---A distances of < 2.7 Å and D-H-A bond angles of > 140º.


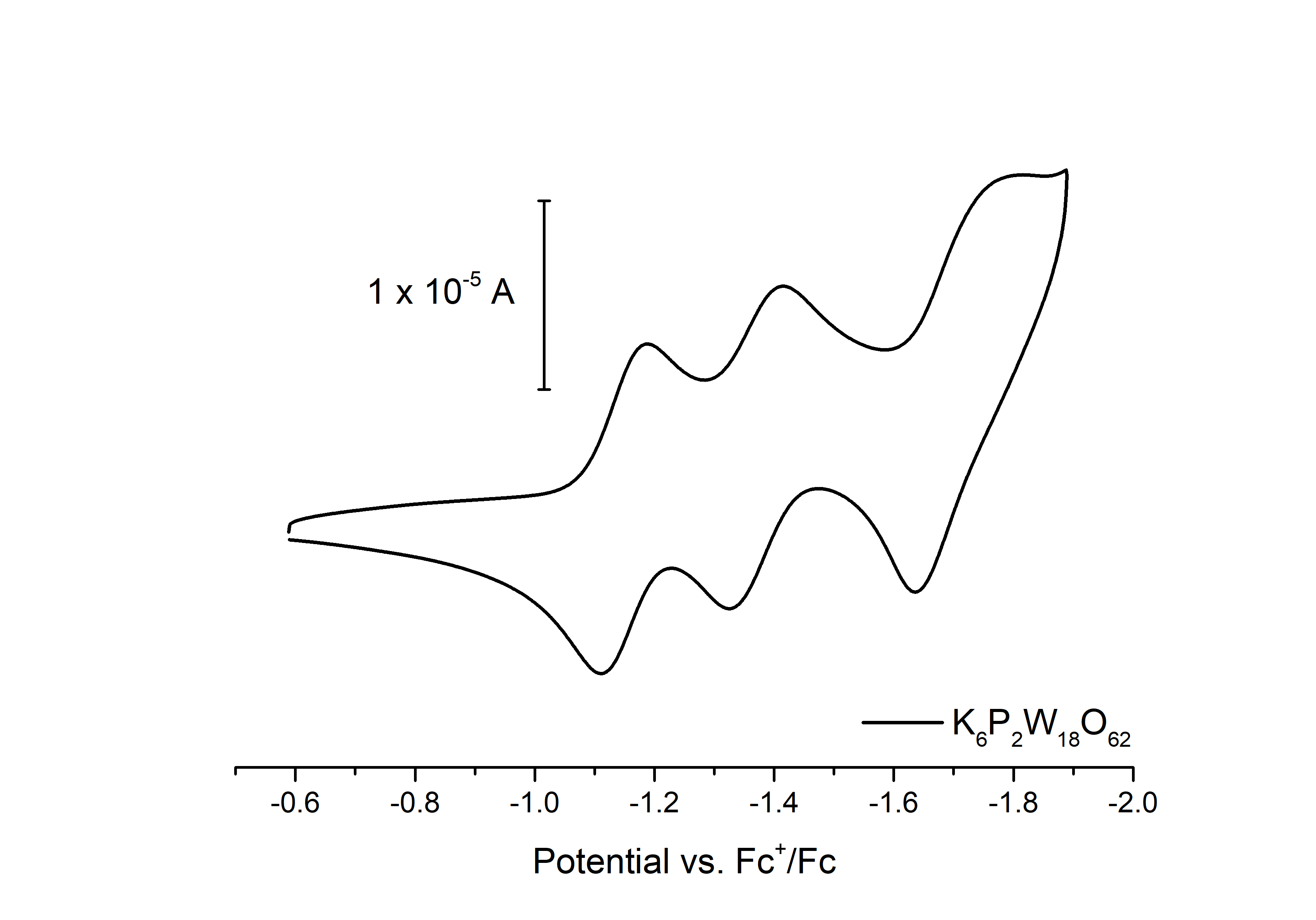


**Figure S8**. Solution-state cyclic voltammetry of K_6_P_2_W_18_O_62_ in DMF with 0.1M TBAPF_6_ as supporting electrolyte. Measurements were performed using a glassy carbon working electrode, platinum counter electrode and silver wire pseudo-reference electrode, with ferrocene added as an internal reference.

**

Figure S9.** Solid-state Fluorescence emission spectra of [BTD-4,7-ImH]Cl_2_ (left) and **1** (right) at various excitation wavelengths; 300 nm (black), 350 nm (red), 400 nm (green), 450 nm (blue).

**Table S1**. Crystal data and structure refinement details for **1**

| Identification code | WAJKLA_sq |
| --- | --- |
| Empirical formula | C_69_H_107_N_29_O_73_P_2_S_3_W_18_ |
| Formula weight | 5978.25 |
| Temperature/K | 120(2) |
| Crystal system | triclinic |
| Space group | P-1 |
| a/Å | 14.0842(6) |
| b/Å | 20.2347(9) |
| c/Å | 21.3071(7) |
| α/° | 91.830(3) |
| β/° | 105.314(4) |
| γ/° | 91.440(4) |
| Volume/Å^3^ | 5850.2(4) |
| Z | 2 |
| ρ_calc_g/cm^3^ | 3.394 |
| μ/mm^‑1^ | 33.488 |
| F(000) | 5436.0 |
| Crystal size/mm^3^ | 0.1 × 0.024 × 0.011 |
| Radiation | CuKα (λ = 1.54184) |
| 2Θ range for data collection/° | 7.718 to 133.202 |
| Index ranges | -16 ≤ h ≤ 8, -24 ≤ k ≤ 23, -25 ≤ l ≤ 25 |
| Reflections collected | 71800 |
| Independent reflections | 20405 [R_int_ = 0.1309, R_sigma_ = 0.1040] |
| Data/restraints/parameters | 20405/2934/1392 |
| Goodness-of-fit on F^2^ | 1.062 |
| Final R indexes [I>=2σ (I)] | R_1_ = 0.0961, wR_2_ = 0.2766 |
| Final R indexes [all data] | R_1_ = 0.1363, wR_2_ = 0.3108 |
| Largest diff. peak/hole / e Å^-3^ | 5.03/-6.08 |

**Table S2**. Bond lengths for **1**

| **Atom** | **Atom** | **Length/Å** |  | **Atom** | **Atom** | **Length/Å** |
| --- | --- | --- | --- | --- | --- | --- |
| W1 | O1 | 1.711(18) |  | W17 | O47 | 1.834(19) |
| W1 | O19 | 1.98(2) |  | W17 | O48 | 1.944(18) |
| W1 | O21 | 1.99(2) |  | W17 | O52 | 1.885(19) |
| W1 | O22 | 1.90(2) |  | W17 | O53 | 1.990(19) |
| W1 | O27 | 1.89(2) |  | W17 | O62 | 2.360(18) |
| W1 | O55 | 2.388(17) |  | W18 | O18 | 1.736(19) |
| W2 | O2 | 1.72(2) |  | W18 | O49 | 1.888(18) |
| W2 | O19 | 1.907(18) |  | W18 | O50 | 1.900(18) |
| W2 | O20 | 1.97(2) |  | W18 | O53 | 1.910(19) |
| W2 | O23 | 1.93(2) |  | W18 | O54 | 1.83(2) |
| W2 | O24 | 1.876(18) |  | W18 | O62 | 2.389(17) |
| W2 | O55 | 2.39(2) |  | P1 | O55 | 1.55(2) |
| W3 | O3 | 1.72(2) |  | P1 | O56 | 1.58(2) |
| W3 | O20 | 1.96(2) |  | P1 | O57 | 1.499(18) |
| W3 | O21 | 1.84(2) |  | P1 | O58 | 1.55(2) |
| W3 | O25 | 1.973(18) |  | P2 | O59 | 1.583(18) |
| W3 | O26 | 1.79(2) |  | P2 | O60 | 1.501(18) |
| W3 | O55 | 2.41(2) |  | P2 | O61 | 1.536(19) |
| W4 | O4 | 1.667(19) |  | P2 | O62 | 1.597(19) |
| W4 | O22 | 1.95(2) |  | S1 | N2 | 1.590(19) |
| W4 | O28 | 1.92(2) |  | S1 | N9 | 1.584(19) |
| W4 | O33 | 1.87(2) |  | N2 | C3 | 1.36(2) |
| W4 | O34 | 1.88(2) |  | N9 | C8 | 1.37(2) |
| W4 | O56 | 2.396(17) |  | N10 | C4 | 1.44(2) |
| W5 | O5 | 1.62(2) |  | N10 | C11 | 1.36(2) |
| W5 | O23 | 1.92(2) |  | N10 | C14 | 1.37(2) |
| W5 | O28 | 1.887(18) |  | N12 | C11 | 1.31(2) |
| W5 | O29 | 1.954(17) |  | N12 | C13 | 1.40(2) |
| W5 | O35 | 1.892(19) |  | N15 | C7 | 1.44(2) |
| W5 | O56 | 2.321(19) |  | N15 | C16 | 1.36(2) |
| W6 | O6 | 1.70(2) |  | N15 | C19 | 1.37(2) |
| W6 | O24 | 2.001(19) |  | N17 | C16 | 1.31(2) |
| W6 | O29 | 1.833(18) |  | N17 | C18 | 1.39(2) |
| W6 | O30 | 1.93(2) |  | C3 | C4 | 1.38(2) |
| W6 | O36 | 1.882(18) |  | C3 | C8 | 1.46(3) |
| W6 | O57 | 2.408(19) |  | C4 | C5 | 1.34(2) |
| W7 | O7 | 1.774(18) |  | C5 | C6 | 1.46(3) |
| W7 | O25 | 1.960(19) |  | C6 | C7 | 1.34(2) |
| W7 | O30 | 1.92(2) |  | C7 | C8 | 1.38(2) |
| W7 | O31 | 1.861(19) |  | C13 | C14 | 1.32(2) |
| W7 | O37 | 1.940(18) |  | C18 | C19 | 1.32(2) |
| W7 | O57 | 2.347(18) |  | S21 | N22 | 1.588(19) |
| W8 | O8 | 1.702(19) |  | S21 | N29 | 1.598(19) |
| W8 | O26 | 2.05(2) |  | N22 | C23 | 1.37(2) |
| W8 | O31 | 1.942(17) |  | N29 | C28 | 1.37(2) |
| W8 | O32 | 1.855(19) |  | N30 | C24 | 1.43(2) |
| W8 | O38 | 1.85(2) |  | N30 | C31 | 1.37(2) |
| W8 | O58 | 2.384(19) |  | N30 | C34 | 1.36(2) |
| W9 | O9 | 1.684(19) |  | N32 | C31 | 1.31(2) |
| W9 | O27 | 1.98(2) |  | N32 | C33 | 1.41(2) |
| W9 | O32 | 1.927(19) |  | N35 | C27 | 1.44(2) |
| W9 | O33 | 1.91(2) |  | N35 | C36 | 1.37(2) |
| W9 | O39 | 1.865(19) |  | N35 | C39 | 1.36(2) |
| W9 | O58 | 2.376(17) |  | N37 | C36 | 1.32(2) |
| W10 | O10 | 1.710(17) |  | N37 | C38 | 1.42(2) |
| W10 | O34 | 1.90(2) |  | C23 | C24 | 1.37(2) |
| W10 | O40 | 1.900(19) |  | C23 | C28 | 1.47(3) |
| W10 | O45 | 1.934(18) |  | C24 | C25 | 1.35(2) |
| W10 | O46 | 2.011(19) |  | C25 | C26 | 1.45(3) |
| W10 | O59 | 2.362(16) |  | C26 | C27 | 1.34(2) |
| W11 | O11 | 1.66(2) |  | C27 | C28 | 1.38(2) |
| W11 | O35 | 1.893(19) |  | C33 | C34 | 1.32(2) |
| W11 | O40 | 1.924(17) |  | C38 | C39 | 1.31(2) |
| W11 | O41 | 1.923(17) |  | S41 | N42 | 1.597(19) |
| W11 | O47 | 2.018(19) |  | S41 | N49 | 1.581(19) |
| W11 | O59 | 2.323(17) |  | N42 | C43 | 1.36(2) |
| W12 | O12 | 1.683(17) |  | N49 | C48 | 1.36(2) |
| W12 | O36 | 1.923(19) |  | N50 | C44 | 1.44(2) |
| W12 | O41 | 1.877(17) |  | N50 | C51 | 1.37(2) |
| W12 | O42 | 1.909(19) |  | N50 | C54 | 1.37(2) |
| W12 | O48 | 1.888(19) |  | N52 | C51 | 1.31(2) |
| W12 | O60 | 2.382(16) |  | N52 | C53 | 1.40(2) |
| W13 | O13 | 1.721(16) |  | N55 | C47 | 1.43(2) |
| W13 | O37 | 1.843(19) |  | N55 | C56 | 1.37(2) |
| W13 | O42 | 1.90(2) |  | N55 | C59 | 1.36(2) |
| W13 | O43 | 1.862(17) |  | N57 | C56 | 1.31(2) |
| W13 | O49 | 1.949(19) |  | N57 | C58 | 1.40(2) |
| W13 | O60 | 2.366(17) |  | C43 | C44 | 1.37(2) |
| W14 | O14 | 1.678(19) |  | C43 | C48 | 1.46(3) |
| W14 | O38 | 1.936(19) |  | C44 | C45 | 1.35(2) |
| W14 | O43 | 1.929(17) |  | C45 | C46 | 1.44(3) |
| W14 | O44 | 1.828(19) |  | C46 | C47 | 1.34(2) |
| W14 | O50 | 1.941(19) |  | C47 | C48 | 1.37(2) |
| W14 | O61 | 2.357(19) |  | C53 | C54 | 1.32(2) |
| W15 | O15 | 1.61(2) |  | C58 | C59 | 1.32(2) |
| W15 | O39 | 1.920(19) |  | O1S | C2S | 1.23(3) |
| W15 | O44 | 1.944(19) |  | N3S | C2S | 1.29(3) |
| W15 | O45 | 1.857(18) |  | N3S | C4S | 1.48(3) |
| W15 | O51 | 1.958(19) |  | N3S | C5S | 1.49(2) |
| W15 | O61 | 2.394(17) |  | O6S | C7S | 1.23(3) |
| W16 | O16 | 1.58(2) |  | N8S | C7S | 1.29(4) |
| W16 | O46 | 1.862(19) |  | N8S | C9S | 1.49(3) |
| W16 | O51 | 1.892(19) |  | N8S | C10S | 1.49(3) |
| W16 | O52 | 1.996(18) |  | O11S | C12S | 1.22(3) |
| W16 | O54 | 2.009(18) |  | N13S | C12S | 1.30(4) |
| W16 | O62 | 2.411(18) |  | N13S | C14S | 1.48(3) |
| W17 | O17 | 1.711(19) |  | N13S | C15S | 1.49(3) |

**Table S3**. Bond angles for **1**

| **Atom** | **Atom** | **Atom** | **Angle/˚** |  | **Atom** | **Atom** | **Atom** | **Angle/˚** |
| --- | --- | --- | --- | --- | --- | --- | --- | --- |
| O1 | W1 | O19 | 100.1(9) |  | O16 | W16 | O52 | 100.2(9) |
| O1 | W1 | O21 | 101.5(10) |  | O16 | W16 | O54 | 104.0(9) |
| O1 | W1 | O22 | 102.3(9) |  | O16 | W16 | O62 | 170.0(8) |
| O1 | W1 | O27 | 102.8(9) |  | O46 | W16 | O51 | 89.8(8) |
| O1 | W1 | O55 | 170.8(9) |  | O46 | W16 | O52 | 87.2(8) |
| O19 | W1 | O21 | 88.4(9) |  | O46 | W16 | O54 | 155.0(8) |
| O19 | W1 | O55 | 73.8(7) |  | O46 | W16 | O62 | 85.1(7) |
| O21 | W1 | O55 | 71.9(7) |  | O51 | W16 | O52 | 156.5(8) |
| O22 | W1 | O19 | 87.4(9) |  | O51 | W16 | O54 | 88.4(8) |
| O22 | W1 | O21 | 156.3(8) |  | O51 | W16 | O62 | 84.8(7) |
| O22 | W1 | O55 | 84.5(7) |  | O52 | W16 | O54 | 84.7(7) |
| O27 | W1 | O19 | 157.2(7) |  | O52 | W16 | O62 | 71.8(7) |
| O27 | W1 | O21 | 85.8(9) |  | O54 | W16 | O62 | 70.0(7) |
| O27 | W1 | O22 | 89.0(9) |  | O17 | W17 | O47 | 103.6(9) |
| O27 | W1 | O55 | 83.4(7) |  | O17 | W17 | O48 | 103.2(8) |
| O2 | W2 | O19 | 100.4(8) |  | O17 | W17 | O52 | 98.8(8) |
| O2 | W2 | O20 | 101.4(10) |  | O17 | W17 | O53 | 99.9(8) |
| O2 | W2 | O23 | 100.7(10) |  | O17 | W17 | O62 | 169.3(8) |
| O2 | W2 | O24 | 100.4(8) |  | O47 | W17 | O48 | 87.2(8) |
| O2 | W2 | O55 | 173.7(8) |  | O47 | W17 | O52 | 89.3(8) |
| O19 | W2 | O20 | 89.0(9) |  | O47 | W17 | O53 | 156.4(8) |
| O19 | W2 | O23 | 87.5(9) |  | O47 | W17 | O62 | 85.0(7) |
| O19 | W2 | O55 | 75.1(7) |  | O48 | W17 | O53 | 86.2(8) |
| O20 | W2 | O55 | 74.4(8) |  | O48 | W17 | O62 | 83.2(7) |
| O23 | W2 | O20 | 158.0(9) |  | O52 | W17 | O48 | 157.9(8) |
| O23 | W2 | O55 | 83.7(8) |  | O52 | W17 | O53 | 88.4(8) |
| O24 | W2 | O19 | 158.9(9) |  | O52 | W17 | O62 | 74.8(7) |
| O24 | W2 | O20 | 89.7(9) |  | O53 | W17 | O62 | 71.8(6) |
| O24 | W2 | O23 | 85.8(8) |  | O18 | W18 | O49 | 102.7(8) |
| O24 | W2 | O55 | 84.3(7) |  | O18 | W18 | O50 | 103.2(8) |
| O3 | W3 | O20 | 100.8(11) |  | O18 | W18 | O53 | 99.9(8) |
| O3 | W3 | O21 | 97.5(10) |  | O18 | W18 | O54 | 101.0(8) |
| O3 | W3 | O25 | 105.4(10) |  | O18 | W18 | O62 | 170.3(8) |
| O3 | W3 | O26 | 100.4(11) |  | O49 | W18 | O50 | 87.4(8) |
| O3 | W3 | O55 | 169.8(9) |  | O49 | W18 | O53 | 86.1(8) |
| O20 | W3 | O25 | 85.5(8) |  | O49 | W18 | O62 | 82.9(7) |
| O20 | W3 | O55 | 74.3(8) |  | O50 | W18 | O53 | 156.8(8) |
| O21 | W3 | O20 | 88.7(9) |  | O50 | W18 | O62 | 84.7(7) |
| O21 | W3 | O25 | 157.0(9) |  | O53 | W18 | O62 | 72.4(7) |
| O21 | W3 | O55 | 73.8(8) |  | O54 | W18 | O49 | 156.2(8) |
| O25 | W3 | O55 | 83.3(7) |  | O54 | W18 | O50 | 90.3(8) |
| O26 | W3 | O20 | 158.9(9) |  | O54 | W18 | O53 | 86.7(8) |
| O26 | W3 | O21 | 88.6(9) |  | O54 | W18 | O62 | 73.2(7) |
| O26 | W3 | O25 | 89.0(8) |  | O55 | P1 | O56 | 107.2(11) |
| O26 | W3 | O55 | 84.8(8) |  | O57 | P1 | O55 | 108.8(10) |
| O4 | W4 | O22 | 96.7(9) |  | O57 | P1 | O56 | 112.0(11) |
| O4 | W4 | O28 | 102.8(9) |  | O57 | P1 | O58 | 110.0(11) |
| O4 | W4 | O33 | 102.0(9) |  | O58 | P1 | O55 | 107.8(11) |
| O4 | W4 | O34 | 99.9(9) |  | O58 | P1 | O56 | 110.9(11) |
| O4 | W4 | O56 | 174.2(9) |  | O59 | P2 | O62 | 105.5(10) |
| O22 | W4 | O56 | 81.8(7) |  | O60 | P2 | O59 | 111.9(10) |
| O28 | W4 | O22 | 88.5(8) |  | O60 | P2 | O61 | 112.6(11) |
| O28 | W4 | O56 | 71.6(7) |  | O60 | P2 | O62 | 107.5(9) |
| O33 | W4 | O22 | 84.4(9) |  | O61 | P2 | O59 | 111.5(9) |
| O33 | W4 | O28 | 154.9(8) |  | O61 | P2 | O62 | 107.4(10) |
| O33 | W4 | O34 | 90.4(8) |  | W2 | O19 | W1 | 120.8(10) |
| O33 | W4 | O56 | 83.5(7) |  | W3 | O20 | W2 | 120.5(10) |
| O34 | W4 | O22 | 163.3(7) |  | W3 | O21 | W1 | 124.3(11) |
| O34 | W4 | O28 | 89.6(8) |  | W1 | O22 | W4 | 149.1(12) |
| O34 | W4 | O56 | 81.8(7) |  | W5 | O23 | W2 | 150.7(10) |
| O5 | W5 | O23 | 95.4(10) |  | W2 | O24 | W6 | 151.1(11) |
| O5 | W5 | O28 | 100.4(9) |  | W7 | O25 | W3 | 145.9(11) |
| O5 | W5 | O29 | 104.0(9) |  | W3 | O26 | W8 | 151.4(11) |
| O5 | W5 | O35 | 99.5(10) |  | W1 | O27 | W9 | 149.0(11) |
| O5 | W5 | O56 | 173.7(8) |  | W5 | O28 | W4 | 123.6(10) |
| O23 | W5 | O29 | 85.3(8) |  | W6 | O29 | W5 | 155.3(11) |
| O23 | W5 | O56 | 82.1(8) |  | W7 | O30 | W6 | 122.3(10) |
| O28 | W5 | O23 | 89.8(8) |  | W7 | O31 | W8 | 154.0(11) |
| O28 | W5 | O29 | 155.5(8) |  | W8 | O32 | W9 | 125.1(10) |
| O28 | W5 | O35 | 90.1(8) |  | W4 | O33 | W9 | 157.6(12) |
| O28 | W5 | O56 | 74.0(7) |  | W4 | O34 | W10 | 162.7(12) |
| O29 | W5 | O56 | 81.6(7) |  | W5 | O35 | W11 | 162.0(11) |
| O35 | W5 | O23 | 164.9(8) |  | W6 | O36 | W12 | 160.6(11) |
| O35 | W5 | O29 | 88.6(8) |  | W13 | O37 | W7 | 163.1(11) |
| O35 | W5 | O56 | 83.3(7) |  | W8 | O38 | W14 | 160.3(11) |
| O6 | W6 | O24 | 94.5(9) |  | W9 | O39 | W15 | 162.4(11) |
| O6 | W6 | O29 | 105.2(10) |  | W10 | O40 | W11 | 122.6(10) |
| O6 | W6 | O30 | 98.2(10) |  | W12 | O41 | W11 | 153.5(11) |
| O6 | W6 | O36 | 99.7(9) |  | W13 | O42 | W12 | 124.5(9) |
| O6 | W6 | O57 | 169.8(9) |  | W13 | O43 | W14 | 154.6(11) |
| O24 | W6 | O57 | 80.9(7) |  | W14 | O44 | W15 | 125.6(10) |
| O29 | W6 | O24 | 84.1(8) |  | W15 | O45 | W10 | 153.7(10) |
| O29 | W6 | O30 | 155.9(8) |  | W16 | O46 | W10 | 147.6(11) |
| O29 | W6 | O36 | 90.9(8) |  | W17 | O47 | W11 | 150.5(11) |
| O29 | W6 | O57 | 83.5(7) |  | W12 | O48 | W17 | 150.1(10) |
| O30 | W6 | O24 | 88.6(8) |  | W18 | O49 | W13 | 149.6(9) |
| O30 | W6 | O57 | 72.7(7) |  | W18 | O50 | W14 | 151.4(11) |
| O36 | W6 | O24 | 165.8(8) |  | W16 | O51 | W15 | 149.0(11) |
| O36 | W6 | O30 | 90.6(8) |  | W17 | O52 | W16 | 122.5(10) |
| O36 | W6 | O57 | 85.3(7) |  | W18 | O53 | W17 | 123.2(8) |
| O7 | W7 | O25 | 93.7(9) |  | W18 | O54 | W16 | 125.8(10) |
| O7 | W7 | O30 | 100.9(9) |  | W1 | O55 | W2 | 90.2(6) |
| O7 | W7 | O31 | 101.0(9) |  | W1 | O55 | W3 | 89.9(6) |
| O7 | W7 | O37 | 101.1(9) |  | W2 | O55 | W3 | 90.7(7) |
| O7 | W7 | O57 | 173.3(8) |  | P1 | O55 | W1 | 125.7(10) |
| O25 | W7 | O57 | 81.5(7) |  | P1 | O55 | W2 | 124.7(11) |
| O30 | W7 | O25 | 86.5(8) |  | P1 | O55 | W3 | 124.7(11) |
| O30 | W7 | O37 | 90.7(8) |  | W5 | O56 | W4 | 90.7(6) |
| O30 | W7 | O57 | 74.3(7) |  | P1 | O56 | W4 | 126.2(10) |
| O31 | W7 | O25 | 87.1(8) |  | P1 | O56 | W5 | 128.7(11) |
| O31 | W7 | O30 | 157.6(8) |  | W7 | O57 | W6 | 90.3(6) |
| O31 | W7 | O37 | 90.0(8) |  | P1 | O57 | W6 | 126.3(11) |
| O31 | W7 | O57 | 83.5(7) |  | P1 | O57 | W7 | 130.6(11) |
| O37 | W7 | O25 | 165.2(8) |  | W9 | O58 | W8 | 89.6(6) |
| O37 | W7 | O57 | 83.8(7) |  | P1 | O58 | W8 | 127.2(11) |
| O8 | W8 | O26 | 99.2(9) |  | P1 | O58 | W9 | 128.5(10) |
| O8 | W8 | O31 | 102.8(8) |  | W11 | O59 | W10 | 91.5(6) |
| O8 | W8 | O32 | 100.5(9) |  | P2 | O59 | W10 | 127.4(9) |
| O8 | W8 | O38 | 96.0(10) |  | P2 | O59 | W11 | 129.2(9) |
| O8 | W8 | O58 | 173.6(8) |  | W13 | O60 | W12 | 90.4(5) |
| O26 | W8 | O58 | 81.7(8) |  | P2 | O60 | W12 | 127.3(10) |
| O31 | W8 | O26 | 83.7(8) |  | P2 | O60 | W13 | 128.2(10) |
| O31 | W8 | O58 | 83.6(7) |  | W14 | O61 | W15 | 89.9(6) |
| O32 | W8 | O26 | 91.1(8) |  | P2 | O61 | W14 | 127.9(9) |
| O32 | W8 | O31 | 156.6(8) |  | P2 | O61 | W15 | 129.5(11) |
| O32 | W8 | O58 | 73.1(7) |  | W17 | O62 | W16 | 91.0(7) |
| O38 | W8 | O26 | 164.6(9) |  | W17 | O62 | W18 | 92.5(6) |
| O38 | W8 | O31 | 90.6(8) |  | W18 | O62 | W16 | 90.9(6) |
| O38 | W8 | O32 | 88.5(8) |  | P2 | O62 | W16 | 123.8(8) |
| O38 | W8 | O58 | 83.5(8) |  | P2 | O62 | W17 | 125.5(10) |
| O9 | W9 | O27 | 96.7(9) |  | P2 | O62 | W18 | 123.3(10) |
| O9 | W9 | O32 | 100.9(10) |  | N9 | S1 | N2 | 99.6(15) |
| O9 | W9 | O33 | 104.2(9) |  | C3 | N2 | S1 | 111(2) |
| O9 | W9 | O39 | 98.3(9) |  | C8 | N9 | S1 | 108(2) |
| O9 | W9 | O58 | 172.6(9) |  | C11 | N10 | C4 | 125(3) |
| O27 | W9 | O58 | 81.0(7) |  | C11 | N10 | C14 | 111(3) |
| O32 | W9 | O27 | 88.9(8) |  | C14 | N10 | C4 | 124(3) |
| O32 | W9 | O58 | 72.2(7) |  | C11 | N12 | C13 | 108(3) |
| O33 | W9 | O27 | 84.0(9) |  | C16 | N15 | C7 | 125(3) |
| O33 | W9 | O32 | 154.6(8) |  | C16 | N15 | C19 | 107(3) |
| O33 | W9 | O58 | 82.6(7) |  | C19 | N15 | C7 | 127(3) |
| O39 | W9 | O27 | 165.0(7) |  | C16 | N17 | C18 | 114(4) |
| O39 | W9 | O32 | 89.4(8) |  | N2 | C3 | C4 | 131(3) |
| O39 | W9 | O33 | 91.2(8) |  | N2 | C3 | C8 | 108(2) |
| O39 | W9 | O58 | 84.3(7) |  | C4 | C3 | C8 | 122(2) |
| O10 | W10 | O34 | 100.4(8) |  | C3 | C4 | N10 | 121(3) |
| O10 | W10 | O40 | 100.4(9) |  | C5 | C4 | N10 | 122(3) |
| O10 | W10 | O45 | 102.5(8) |  | C5 | C4 | C3 | 117(3) |
| O10 | W10 | O46 | 93.8(8) |  | C4 | C5 | C6 | 126(4) |
| O10 | W10 | O59 | 171.9(8) |  | C7 | C6 | C5 | 113(3) |
| O34 | W10 | O40 | 91.5(8) |  | C6 | C7 | N15 | 115(3) |
| O34 | W10 | O45 | 89.0(8) |  | C6 | C7 | C8 | 127(3) |
| O34 | W10 | O46 | 165.8(7) |  | C8 | C7 | N15 | 118(3) |
| O34 | W10 | O59 | 83.9(7) |  | N9 | C8 | C3 | 114(2) |
| O40 | W10 | O45 | 156.6(7) |  | N9 | C8 | C7 | 131(3) |
| O40 | W10 | O46 | 87.7(8) |  | C7 | C8 | C3 | 115(2) |
| O40 | W10 | O59 | 72.5(7) |  | N12 | C11 | N10 | 106(3) |
| O45 | W10 | O46 | 86.2(8) |  | C14 | C13 | N12 | 110(3) |
| O45 | W10 | O59 | 84.3(6) |  | C13 | C14 | N10 | 105(3) |
| O46 | W10 | O59 | 82.3(7) |  | N17 | C16 | N15 | 105(3) |
| O11 | W11 | O35 | 99.7(9) |  | C19 | C18 | N17 | 102(4) |
| O11 | W11 | O40 | 99.4(9) |  | C18 | C19 | N15 | 112(4) |
| O11 | W11 | O41 | 103.6(9) |  | N22 | S21 | N29 | 104.1(15) |
| O11 | W11 | O47 | 95.4(8) |  | C23 | N22 | S21 | 106.4(19) |
| O11 | W11 | O59 | 171.8(8) |  | C28 | N29 | S21 | 104.4(19) |
| O35 | W11 | O40 | 90.9(8) |  | C31 | N30 | C24 | 131(2) |
| O35 | W11 | O41 | 90.9(8) |  | C34 | N30 | C24 | 124(2) |
| O35 | W11 | O47 | 164.8(8) |  | C34 | N30 | C31 | 105(2) |
| O35 | W11 | O59 | 83.9(7) |  | C31 | N32 | C33 | 108(3) |
| O40 | W11 | O47 | 88.0(7) |  | C36 | N35 | C27 | 128(3) |
| O40 | W11 | O59 | 73.0(7) |  | C39 | N35 | C27 | 125(2) |
| O41 | W11 | O40 | 156.3(8) |  | C39 | N35 | C36 | 107(2) |
| O41 | W11 | O47 | 84.1(7) |  | C36 | N37 | C38 | 101(3) |
| O41 | W11 | O59 | 83.7(7) |  | N22 | C23 | C24 | 128(2) |
| O47 | W11 | O59 | 81.3(7) |  | N22 | C23 | C28 | 111(2) |
| O12 | W12 | O36 | 97.9(9) |  | C24 | C23 | C28 | 121(2) |
| O12 | W12 | O41 | 102.8(9) |  | C23 | C24 | N30 | 120(2) |
| O12 | W12 | O42 | 101.0(9) |  | C25 | C24 | N30 | 122(2) |
| O12 | W12 | O48 | 97.5(9) |  | C25 | C24 | C23 | 118(3) |
| O12 | W12 | O60 | 173.2(8) |  | C24 | C25 | C26 | 122(3) |
| O36 | W12 | O60 | 82.3(7) |  | C27 | C26 | C25 | 119(3) |
| O41 | W12 | O36 | 88.8(8) |  | C26 | C27 | N35 | 116(2) |
| O41 | W12 | O42 | 156.1(7) |  | C26 | C27 | C28 | 121(3) |
| O41 | W12 | O48 | 86.2(8) |  | C28 | C27 | N35 | 122(3) |
| O41 | W12 | O60 | 84.0(7) |  | N29 | C28 | C23 | 114(2) |
| O42 | W12 | O36 | 89.2(8) |  | N29 | C28 | C27 | 127(3) |
| O42 | W12 | O60 | 72.2(7) |  | C27 | C28 | C23 | 118(2) |
| O48 | W12 | O36 | 164.5(7) |  | N32 | C31 | N30 | 110(3) |
| O48 | W12 | O42 | 89.4(8) |  | C34 | C33 | N32 | 106(3) |
| O48 | W12 | O60 | 82.6(7) |  | C33 | C34 | N30 | 111(3) |
| O13 | W13 | O37 | 99.2(9) |  | N37 | C36 | N35 | 113(3) |
| O13 | W13 | O42 | 102.4(9) |  | C39 | C38 | N37 | 113(3) |
| O13 | W13 | O43 | 101.3(9) |  | C38 | C39 | N35 | 106(3) |
| O13 | W13 | O49 | 97.6(8) |  | N49 | S41 | N42 | 97.1(17) |
| O13 | W13 | O60 | 174.9(8) |  | C43 | N42 | S41 | 111(2) |
| O37 | W13 | O42 | 91.1(9) |  | C48 | N49 | S41 | 109(2) |
| O37 | W13 | O43 | 88.8(8) |  | C51 | N50 | C44 | 126(3) |
| O37 | W13 | O49 | 163.1(7) |  | C54 | N50 | C44 | 125(3) |
| O37 | W13 | O60 | 82.6(7) |  | C54 | N50 | C51 | 109(3) |
| O42 | W13 | O49 | 87.3(8) |  | C51 | N52 | C53 | 116(3) |
| O42 | W13 | O60 | 72.8(6) |  | C56 | N55 | C47 | 127(2) |
| O43 | W13 | O42 | 156.1(7) |  | C59 | N55 | C47 | 125(2) |
| O43 | W13 | O49 | 85.9(8) |  | C59 | N55 | C56 | 107(2) |
| O43 | W13 | O60 | 83.5(7) |  | C56 | N57 | C58 | 110(2) |
| O49 | W13 | O60 | 80.9(7) |  | N42 | C43 | C44 | 129(3) |
| O14 | W14 | O38 | 97.7(9) |  | N42 | C43 | C48 | 107(2) |
| O14 | W14 | O43 | 102.8(8) |  | C44 | C43 | C48 | 123(2) |
| O14 | W14 | O44 | 99.9(9) |  | C43 | C44 | N50 | 121(3) |
| O14 | W14 | O50 | 96.2(9) |  | C45 | C44 | N50 | 120(3) |
| O14 | W14 | O61 | 173.0(8) |  | C45 | C44 | C43 | 119(3) |
| O38 | W14 | O50 | 165.9(8) |  | C44 | C45 | C46 | 118(3) |
| O38 | W14 | O61 | 84.7(7) |  | C47 | C46 | C45 | 124(3) |
| O43 | W14 | O38 | 91.8(8) |  | C46 | C47 | N55 | 120(2) |
| O43 | W14 | O50 | 83.5(8) |  | C46 | C47 | C48 | 118(3) |
| O43 | W14 | O61 | 83.6(7) |  | C48 | C47 | N55 | 121(2) |
| O44 | W14 | O38 | 90.1(8) |  | N49 | C48 | C43 | 112(2) |
| O44 | W14 | O43 | 156.7(8) |  | N49 | C48 | C47 | 128(3) |
| O44 | W14 | O50 | 89.0(8) |  | C47 | C48 | C43 | 118(2) |
| O44 | W14 | O61 | 73.5(7) |  | N52 | C51 | N50 | 102(3) |
| O50 | W14 | O61 | 81.7(7) |  | C54 | C53 | N52 | 101(3) |
| O15 | W15 | O39 | 98.7(9) |  | C53 | C54 | N50 | 111(3) |
| O15 | W15 | O44 | 105.1(9) |  | N57 | C56 | N55 | 107(2) |
| O15 | W15 | O45 | 101.6(9) |  | C59 | C58 | N57 | 105(3) |
| O15 | W15 | O51 | 95.9(9) |  | C58 | C59 | N55 | 110(2) |
| O15 | W15 | O61 | 174.7(8) |  | C2S | N3S | C4S | 124(3) |
| O39 | W15 | O44 | 91.2(8) |  | C2S | N3S | C5S | 119(3) |
| O39 | W15 | O51 | 165.3(7) |  | C4S | N3S | C5S | 117(3) |
| O39 | W15 | O61 | 84.9(6) |  | O1S | C2S | N3S | 126(4) |
| O44 | W15 | O51 | 87.1(8) |  | C7S | N8S | C9S | 128(4) |
| O44 | W15 | O61 | 70.7(7) |  | C7S | N8S | C10S | 118(4) |
| O45 | W15 | O39 | 87.7(8) |  | C10S | N8S | C9S | 112(4) |
| O45 | W15 | O44 | 153.1(7) |  | O6S | C7S | N8S | 119(4) |
| O45 | W15 | O51 | 87.2(8) |  | C12S | N13S | C14S | 124(4) |
| O45 | W15 | O61 | 82.4(7) |  | C12S | N13S | C15S | 120(4) |
| O51 | W15 | O61 | 80.7(7) |  | C14S | N13S | C15S | 115(4) |
| O16 | W16 | O46 | 100.7(9) |  | O11S | C12S | N13S | 123(5) |
| O16 | W16 | O51 | 103.3(9) |  |  |  |  |  |

**Table S4**. Hydrogen bonds for **1**

| **D** | **H** | **A** | **d(D-H)/Å** | **d(H-A)/Å** | **d(D-A)/Å** | **D-H-A/°** |
| --- | --- | --- | --- | --- | --- | --- |
| N12 | H12 | O11S^1^ | 0.88 | 1.66 | 2.54(5) | 176.7 |
| N17 | H17 | O6S^2^ | 0.88 | 1.81 | 2.67(6) | 165.3 |
| C11 | H11 | O2 | 0.95 | 2.11 | 3.06(4) | 176.6 |
| C11 | H11 | N2 | 0.95 | 2.71 | 3.05(4) | 101.9 |
| C16 | H16 | N9 | 0.95 | 2.40 | 2.86(4) | 109.3 |
| C18 | H18 | O8^3^ | 0.95 | 2.61 | 3.09(4) | 111.5 |
| N32 | H32 | O12^4^ | 0.88 | 2.70 | 3.20(3) | 117.6 |
| C25 | H25 | O10 | 0.95 | 2.64 | 2.99(4) | 102.6 |
| C25 | H25 | O47^5^ | 0.95 | 2.84 | 3.14(4) | 99.5 |
| C26 | H26 | O46^5^ | 0.95 | 2.77 | 3.11(4) | 102.0 |
| C31 | H31 | N22 | 0.95 | 2.58 | 2.99(4) | 106.7 |
| C36 | H36 | O5 | 0.95 | 2.33 | 3.11(4) | 138.3 |
| C36 | H36 | N29 | 0.95 | 2.56 | 3.01(4) | 109.7 |
| C38 | H38 | O1S^5^ | 0.95 | 1.88 | 2.77(4) | 156.7 |
| C39 | H39 | O10^5^ | 0.95 | 2.23 | 3.11(4) | 153.9 |
| C39 | H39 | O46^5^ | 0.95 | 2.45 | 3.18(3) | 134.4 |
| N57 | H57 | O13^4^ | 0.88 | 2.49 | 3.01(3) | 118.2 |
| C45 | H45 | O44 | 0.95 | 2.73 | 3.04(4) | 99.4 |
| C46 | H46 | O44 | 0.95 | 2.96 | 3.16(3) | 93.0 |
| C46 | H46 | O50^6^ | 0.95 | 2.83 | 3.08(4) | 96.5 |
| C51 | H51 | N42 | 0.95 | 2.36 | 2.88(4) | 114.0 |
| C54 | H54 | O14 | 0.95 | 2.55 | 3.03(4) | 111.6 |
| C56 | H56 | O13^4^ | 0.95 | 2.52 | 3.02(3) | 113.4 |
| C56 | H56 | N49 | 0.95 | 2.40 | 2.93(3) | 114.8 |
| C59 | H59 | O14^6^ | 0.95 | 2.24 | 3.16(3) | 163.5 |
| C2S | H2S | O15 | 0.95 | 2.37 | 3.15(5) | 139.2 |
| C5S | H5SA | O53^4^ | 0.98 | 2.62 | 2.84(3) | 92.7 |
| C9S | H9SA | O7 | 0.98 | 2.30 | 3.11(6) | 139.4 |
| C10S | H10B | O13 | 0.98 | 2.73 | 2.99(4) | 95.7 |
| C15S | H15A | O11S | 0.98 | 2.32 | 2.72(6) | 103.6 |
| C15S | H15B | O1^7^ | 0.98 | 2.64 | 2.94(6) | 97.8 |

^1^1-X, 1-Y, 2-Z; ^2^1-X, -Y, 2-Z; ^3^ -X, -Y, 2-Z; ^4^ -1+X, +Y, +Z; ^5^ -X, 1-Y, 1-Z; ^6^ -X, -Y, 1-Z; ^7^ 1+X, +Y, +Z

*All H atoms were geometrically located at calculated positions and refined using a riding model

**Table S5**. Selected torsion angles between imidazolium and benzothiadiazoles for **1**

| **A** | **B** | **C** | **D** | **Angle (º)** |
| --- | --- | --- | --- | --- |
| C5 | C4 | N10 | C14 | 35(6) |
| C6 | C7 | N15 | C19 | -34(6) |
| C25 | C24 | N30 | C34 | -23(5) |
| C26 | C27 | N35 | C39 | 20(5) |
| C45 | C44 | N50 | C54 | -3(5) |
| C46 | C47 | N55 | C59 | 19(5) |
