# Supplementary material for: A Cooperative Photoactive Class-I Hybrid Polyoxometalate With Benzothiadiazole–Imidazolium Cations
Source: Front Chem. 2021 Jan 14;8:612535. doi: 10.3389/fchem.2020.612535 (PMC7841050; doi:10.3389/fchem.2020.612535)

checkCIF/PLATON report

Structure factors have been supplied for datablock(s) I

THIS REPORT IS FOR GUIDANCE ONLY. IF USED AS PART OF A REVIEW PROCEDURE FOR PUBLICATION, IT SHOULD NOT REPLACE THE EXPERTISE OF AN EXPERIENCED CRYSTALLOGRAPHIC REFEREE.

No syntax errors found.   CIF dictionary   Interpreting this report

Datablock: I

Bond precision: C-C = 0.0433 A   Wavelength=1.54184

Cell:	a=14.0842(6)	b=20.2347(9)	c=21.3071(7)
	alpha=91.830(3)	beta=105.314(4)	gamma=91.440(4)
Temperature:	120 K		

	Calculated	Reported
Volume	5850.2(4)	5850.2(4)
Space group	P -1	P -1
Hall group	-P 1	-P 1
Moiety formula	O62 P2 W18, 3(C12 H10 N6 S), 3(C3 H7 N O) [+ solvent]	O62 P2 W18, 3(C12 H10 N6 S), 3(C3 H7 N O)
Sum formula	C45 H51 N21 O65 P2 S3 W18 [+ solvent]	C69 H107 N29 O73 P2 S3 W18
Mr	5393.31	5978.25
Dx,g cm-3	3.062	3.394
Z	2	2
Mu (mm-1)	33.282	33.488
F000	4796.0	5436.0
F000'	4614.21	
h,k,lmax	16,24,25	16,24,25
Nref	20679	20405
Tmin,Tmax	0.438,0.692	0.202,0.724
Tmin'	0.031	

Correction method= # Reported T Limits: Tmin=0.202 Tmax=0.724
AbsCorr = GAUSSIAN

Data completeness= 0.987   Theta(max)= 66.601

R(reflections)= 0.0961(12360)   wR2(reflections)= 0.3108(20405)

S = 1.062   Npar= 1392

The following ALERTS were generated. Each ALERT has the format
test-name_ALERT_alert-type_alert-level.
Click on the hyperlinks for more details of the test.

Alert level A

```
PLAT973_ALERT_2_A Check Calcd Positive Resid. Density on       W11       3.46 eA-3
```

Author Response: The X-ray diffraction intensity of the large anionic polyoxometalate was weak and dropped off at high angle with a diffraction limit of 1.1 A. Many large residual electron density peaks are located close to the tungsten atoms (range 0.9-1.9 A) likely as a result of deficiencies in the absorption correction and ellipsoid model for the atomic displacement.

```
PLAT973_ALERT_2_A Check Calcd Positive Resid. Density on        W6       3.14 eA-3
```

Author Response: The X-ray diffraction intensity of the large anionic polyoxometalate was weak and dropped off at high angle with a diffraction limit of 1.1 A. Many large residual electron density peaks are located close to the tungsten atoms (range 0.9-1.9 A) likely as a result of deficiencies in the absorption correction and ellipsoid model for the atomic displacement.

Alert level B

```
PLAT342_ALERT_3_B Low Bond Precision on  C-C Bonds ...............    0.04333 Ang.
```

Author Response: The X-ray diffraction intensity of the large anionic polyoxometalate was weak and dropped off at high angle with a diffraction limit of 1.1 A. Geometric similarity restraints were applied to bond distances of the dimethylformamide solvents and 4,7-imidazolium-2,1,3-benzothiadiazole cations (SADI). Rigid bond and similarity restraints were applied to the anisotropic displacement parameters of all atoms in the structure.

```
PLAT990_ALERT_1_B Deprecated .res/.hkl Input Style SQUEEZE Job ...          ! Note
```

Alert level C

```
DIFMN02_ALERT_2_C  The minimum difference density is < -0.1*ZMAX*0.75
            _refine_diff_density_min given =     -6.084
            Test value =     -5.550
DIFMN03_ALERT_1_C  The minimum difference density is < -0.1*ZMAX*0.75
            The relevant atom site should be identified.
RINTA01_ALERT_3_C  The value of Rint is greater than 0.12
            Rint given   0.131
PLAT020_ALERT_3_C The Value of Rint is Greater Than 0.12 .........      0.131 Report
PLAT084_ALERT_3_C High wR2 Value (i.e. > 0.25) ...................       0.31 Report
PLAT098_ALERT_2_C Large Reported Min.  (Negative) Residual Density      -6.08 eA-3
PLAT234_ALERT_4_C Large Hirshfeld Difference W16      --O16      .       0.17 Ang.
PLAT260_ALERT_2_C Large Average Ueq of Residue Including       O6S      0.119 Check
PLAT260_ALERT_2_C Large Average Ueq of Residue Including      O11S      0.115 Check
PLAT420_ALERT_2_C D-H Without Acceptor       N37      --H37      .     Please Check
PLAT420_ALERT_2_C D-H Without Acceptor       N52      --H52      .     Please Check
```

```
PLAT480_ALERT_4_C Long H...A H-Bond Reported H11      ..N2       .       2.71 Ang.
PLAT480_ALERT_4_C Long H...A H-Bond Reported H18      ..O8       .       2.61 Ang.
PLAT480_ALERT_4_C Long H...A H-Bond Reported H32      ..O12      .       2.70 Ang.
PLAT480_ALERT_4_C Long H...A H-Bond Reported H25      ..O10      .       2.64 Ang.
PLAT480_ALERT_4_C Long H...A H-Bond Reported H25      ..O47      .       2.84 Ang.
PLAT480_ALERT_4_C Long H...A H-Bond Reported H26      ..O46      .       2.77 Ang.
PLAT480_ALERT_4_C Long H...A H-Bond Reported H45      ..O44      .       2.73 Ang.
PLAT480_ALERT_4_C Long H...A H-Bond Reported H46      ..O44      .       2.96 Ang.
PLAT480_ALERT_4_C Long H...A H-Bond Reported H46      ..O50      .       2.83 Ang.
PLAT480_ALERT_4_C Long H...A H-Bond Reported H5SA     ..O53      .       2.62 Ang.
PLAT480_ALERT_4_C Long H...A H-Bond Reported H10B     ..O13      .       2.73 Ang.
PLAT480_ALERT_4_C Long H...A H-Bond Reported H15B     ..O1       .       2.64 Ang.
PLAT482_ALERT_4_C Small D-H..A Angle Rep for C25      ..O47      .      99.50 Degree
PLAT482_ALERT_4_C Small D-H..A Angle Rep for C45      ..O44      .      99.40 Degree
PLAT482_ALERT_4_C Small D-H..A Angle Rep for C46      ..O44      .      93.00 Degree
PLAT482_ALERT_4_C Small D-H..A Angle Rep for C46      ..O50      .      96.50 Degree
PLAT482_ALERT_4_C Small D-H..A Angle Rep for C5S      ..O53      .      92.70 Degree
PLAT482_ALERT_4_C Small D-H..A Angle Rep for C10S     ..O13      .      95.70 Degree
PLAT482_ALERT_4_C Small D-H..A Angle Rep for C15S     ..O1       .      97.80 Degree
PLAT910_ALERT_3_C Missing # of FCF Reflection(s) Below Theta(Min).          6 Note
PLAT911_ALERT_3_C Missing FCF Refl Between Thmin & STh/L=    0.595        268 Report
PLAT923_ALERT_1_C S    Values in the CIF and FCF Differ by .......      0.011 Check
PLAT977_ALERT_2_C Check Negative Difference Density on H11              -0.65 eA-3
PLAT977_ALERT_2_C Check Negative Difference Density on H12              -0.74 eA-3
PLAT977_ALERT_2_C Check Negative Difference Density on H31              -0.43 eA-3
PLAT977_ALERT_2_C Check Negative Difference Density on H33              -0.60 eA-3
PLAT977_ALERT_2_C Check Negative Difference Density on H37              -0.54 eA-3
PLAT977_ALERT_2_C Check Negative Difference Density on H38              -0.55 eA-3
PLAT977_ALERT_2_C Check Negative Difference Density on H45              -0.60 eA-3
PLAT977_ALERT_2_C Check Negative Difference Density on H53              -0.59 eA-3
PLAT977_ALERT_2_C Check Negative Difference Density on H56              -0.60 eA-3
```

Alert level G

```
FORMU01_ALERT_1_G  There is a discrepancy between the atom counts in the
            _chemical_formula_sum and _chemical_formula_moiety. This is
            usually due to the moiety formula being in the wrong format.
            Atom count from _chemical_formula_sum:   C69 H107 N29 O73 P2 S3 W18
            Atom count from _chemical_formula_moiety:C45 H51 N21 O65 P2 S3 W18
FORMU01_ALERT_2_G  There is a discrepancy between the atom counts in the
            _chemical_formula_sum and the formula from the _atom_site* data.
            Atom count from _chemical_formula_sum:C69 H107 N29 O73 P2 S3 W18
            Atom count from the _atom_site data:  C45 H51 N21 O65 P2 S3 W18
CELLZ01_ALERT_1_G Difference between formula and atom_site contents detected.
CELLZ01_ALERT_1_G ALERT: Large difference may be due to a
            symmetry error - see SYMMG tests
           From the CIF: _cell_formula_units_Z    2
           From the CIF: _chemical_formula_sum  C69 H107 N29 O73 P2 S3 W18
           TEST: Compare cell contents of formula and atom_site data

           atom    Z*formula  cif sites diff
           C        138.00     90.00   48.00
           H        214.00    102.00  112.00
           N         58.00     42.00   16.00
           O        146.00    130.00   16.00
           P          4.00      4.00    0.00
           S          6.00      6.00    0.00
           W         36.00     36.00    0.00
PLAT002_ALERT_2_G Number of Distance or Angle Restraints on AtSite         72 Note
PLAT003_ALERT_2_G Number of Uiso or Uij Restrained non-H Atoms ...        154 Report
PLAT005_ALERT_5_G No Embedded Refinement Details Found  in the CIF     Please Do !
```

```
PLAT007_ALERT_5_G Number of Unrefined Donor-H Atoms ..............          6 Report
PLAT041_ALERT_1_G Calc. and Reported SumFormula    Strings  Differ     Please Check
PLAT044_ALERT_1_G Calculated and Reported Density Dx  Differ by ..     0.3323 Check
PLAT051_ALERT_1_G Mu(calc) and Mu(CIF) Ratio Differs from 1.0 by .       0.61 %
PLAT068_ALERT_1_G Reported F000 Differs from Calcd (or Missing)...     Please Check
PLAT072_ALERT_2_G SHELXL First  Parameter in WGHT  Unusually Large       0.20 Report
PLAT380_ALERT_4_G Incorrectly? Oriented X(sp2)-Methyl Moiety .....        C5S Check
PLAT380_ALERT_4_G Incorrectly? Oriented X(sp2)-Methyl Moiety .....        C9S Check
PLAT432_ALERT_2_G Short Inter X...Y Contact  S21      ..C14              3.30 Ang.
                                                -x,1-y,2-z  =      2_567 Check
PLAT432_ALERT_2_G Short Inter X...Y Contact  O1       ..C6               2.85 Ang.
                                                     x,y,z  =      1_555 Check
PLAT432_ALERT_2_G Short Inter X...Y Contact  O1       ..C15S             2.94 Ang.
                                                  -1+x,y,z  =      1_455 Check
PLAT432_ALERT_2_G Short Inter X...Y Contact  O1S      ..C38              2.78 Ang.
                                                -x,1-y,1-z  =      2_566 Check
PLAT432_ALERT_2_G Short Inter X...Y Contact  O8       ..C51              2.80 Ang.
                                                     x,y,z  =      1_555 Check
PLAT432_ALERT_2_G Short Inter X...Y Contact  O10      ..C25              2.99 Ang.
                                                     x,y,z  =      1_555 Check
PLAT432_ALERT_2_G Short Inter X...Y Contact  O13      ..C10S             2.98 Ang.
                                                     x,y,z  =      1_555 Check
PLAT432_ALERT_2_G Short Inter X...Y Contact  O13      ..C56              3.02 Ang.
                                                   1+x,y,z  =      1_655 Check
PLAT432_ALERT_2_G Short Inter X...Y Contact  O40      ..C26              2.97 Ang.
                                                     x,y,z  =      1_555 Check
PLAT432_ALERT_2_G Short Inter X...Y Contact  O53      ..C5S              2.84 Ang.
                                                   1+x,y,z  =      1_655 Check
PLAT606_ALERT_4_G Solvent Accessible VOID(S) in Structure ........          ! Info
PLAT720_ALERT_4_G Number of Unusual/Non-Standard Labels ..........          9 Note
PLAT722_ALERT_1_G Angle   Calc      128.00, Rep      129.20 Dev...       1.20 Degree
              N17     -C18     -H18        1.555  1.555  1.555     #  394 Check
PLAT722_ALERT_1_G Angle   Calc      108.00, Rep      109.50 Dev...       1.50 Degree
              N8S     -C10S    -H10B       1.555  1.555  1.555     #  533 Check
PLAT722_ALERT_1_G Angle   Calc      111.00, Rep      109.50 Dev...       1.50 Degree
              H10A    -C10S    -H10C       1.555  1.555  1.555     #  536 Check
PLAT790_ALERT_4_G Centre of Gravity not Within Unit Cell: Resd.  #          4 Note
              C12 H10 N6 S
PLAT790_ALERT_4_G Centre of Gravity not Within Unit Cell: Resd.  #          5 Note
              C3 H7 N O
PLAT794_ALERT_5_G Tentative Bond Valency for W1        (VI)      .       5.81 Info
PLAT794_ALERT_5_G Tentative Bond Valency for W2        (VI)      .       5.92 Info
PLAT794_ALERT_5_G Tentative Bond Valency for W3        (VI)      .       6.36 Info
PLAT794_ALERT_5_G Tentative Bond Valency for W4        (VI)      .       6.37 Info
PLAT794_ALERT_5_G Tentative Bond Valency for W5        (VI)      .       6.61 Info
PLAT794_ALERT_5_G Tentative Bond Valency for W6        (VI)      .       6.18 Info
PLAT794_ALERT_5_G Tentative Bond Valency for W7        (VI)      .       5.75 Info
PLAT794_ALERT_5_G Tentative Bond Valency for W8        (VI)      .       6.07 Info
PLAT794_ALERT_5_G Tentative Bond Valency for W9        (VI)      .       6.17 Info
PLAT794_ALERT_5_G Tentative Bond Valency for W10       (VI)      .       5.88 Info
PLAT794_ALERT_5_G Tentative Bond Valency for W11       (VI)      .       6.13 Info
PLAT794_ALERT_5_G Tentative Bond Valency for W12       (VI)      .       6.36 Info
PLAT794_ALERT_5_G Tentative Bond Valency for W13       (VI)      .       6.34 Info
PLAT794_ALERT_5_G Tentative Bond Valency for W14       (VI)      .       6.32 Info
PLAT794_ALERT_5_G Tentative Bond Valency for W15       (VI)      .       6.55 Info
PLAT794_ALERT_5_G Tentative Bond Valency for W16       (VI)      .       6.56 Info
PLAT794_ALERT_5_G Tentative Bond Valency for W17       (VI)      .       6.13 Info
PLAT794_ALERT_5_G Tentative Bond Valency for W18       (VI)      .       6.32 Info
PLAT860_ALERT_3_G Number of Least-Squares Restraints .............       2934 Note
PLAT869_ALERT_4_G ALERTS Related to the Use of SQUEEZE Suppressed           ! Info
PLAT909_ALERT_3_G Percentage of I>2sig(I) Data at Theta(Max) Still        33% Note
PLAT978_ALERT_2_G Number C-C Bonds with Positive Residual Density.          0 Info
```

```
  2 ALERT level A = Most likely a serious problem - resolve or explain
  2 ALERT level B = A potentially serious problem, consider carefully
 42 ALERT level C = Check. Ensure it is not caused by an omission or oversight
 54 ALERT level G = General information/check it is not something unexpected

 13 ALERT type 1 CIF construction/syntax error, inconsistent or missing data
 32 ALERT type 2 Indicator that the structure model may be wrong or deficient
  8 ALERT type 3 Indicator that the structure quality may be low
 27 ALERT type 4 Improvement, methodology, query or suggestion
 20 ALERT type 5 Informative message, check
```

checkCIF publication errors

Alert level A

```
PUBL004_ALERT_1_A  The contact author's name and address are missing,
            _publ_contact_author_name and _publ_contact_author_address.
PUBL005_ALERT_1_A  _publ_contact_author_email, _publ_contact_author_fax and
            _publ_contact_author_phone are all missing.
            At least one of these should be present.
PUBL006_ALERT_1_A  _publ_requested_journal is missing
            e.g. 'Acta Crystallographica Section C'
PUBL008_ALERT_1_A  _publ_section_title is missing. Title of paper.
PUBL009_ALERT_1_A  _publ_author_name is missing. List of author(s) name(s).
PUBL010_ALERT_1_A  _publ_author_address is missing. Author(s) address(es).
PUBL012_ALERT_1_A  _publ_section_abstract is missing.
            Abstract of paper in English.
```

```
  7 ALERT level A = Data missing that is essential or data in wrong format
  0 ALERT level G = General alerts. Data that may be required is missing
```

Publication of your CIF

You should attempt to resolve as many as possible of the alerts in all categories. Often the minor alerts point to easily fixed oversights, errors and omissions in your CIF or refinement strategy, so attention to these fine details can be worthwhile. In order to resolve some of the more serious problems it may be necessary to carry out additional measurements or structure refinements. However, the nature of your study may justify the reported deviations from journal submission requirements and the more serious of these should be commented upon in the discussion or experimental section of a paper or in the "special_details" fields of the CIF. *checkCIF* was carefully designed to identify outliers and unusual parameters, but every test has its limitations and alerts that are not important in a particular case may appear. Conversely, the absence of alerts does not guarantee there are no aspects of the results needing attention. It is up to the individual to critically assess their own results and, if necessary, seek expert advice.

If level A alerts remain, which you believe to be justified deviations, and you intend to submit this CIF for publication in a journal, you should additionally insert an explanation in your CIF using the Validation Reply Form (VRF) below. This will allow your explanation to be considered as part of the review process.

Validation response form

Please find below a validation response form (VRF) that can be filled in and pasted into your CIF.

```
# start Validation Reply Form
_vrf_PUBL004_GLOBAL
;
PROBLEM: The contact author’s name and address are missing,
RESPONSE: ...
;
_vrf_PUBL005_GLOBAL
;
PROBLEM: _publ_contact_author_email, _publ_contact_author_fax and
RESPONSE: ...
;
_vrf_PUBL006_GLOBAL
;
PROBLEM: _publ_requested_journal is missing
RESPONSE: ...
;
_vrf_PUBL008_GLOBAL
;
PROBLEM: _publ_section_title is missing. Title of paper.
RESPONSE: ...
;
_vrf_PUBL009_GLOBAL
;
PROBLEM: _publ_author_name is missing. List of author(s) name(s).
RESPONSE: ...
;
_vrf_PUBL010_GLOBAL
;
PROBLEM: _publ_author_address is missing. Author(s) address(es).
RESPONSE: ...
;
_vrf_PUBL012_GLOBAL
;
PROBLEM: _publ_section_abstract is missing.
RESPONSE: ...
;
# end Validation Reply Form
```

If you wish to submit your CIF for publication in Acta Crystallographica Section C or E, you should upload your CIF via the web. If you wish to submit your CIF for publication in IUCrData you should upload your CIF via the web. If your CIF is to form part of a submission to another IUCr journal, you will be asked, either during electronic submission or by the Co-editor handling your paper, to upload your CIF via our web site.

PLATON version of 10/08/2020; check.def file version of 06/08/2020

Datablock I - ellipsoid plot

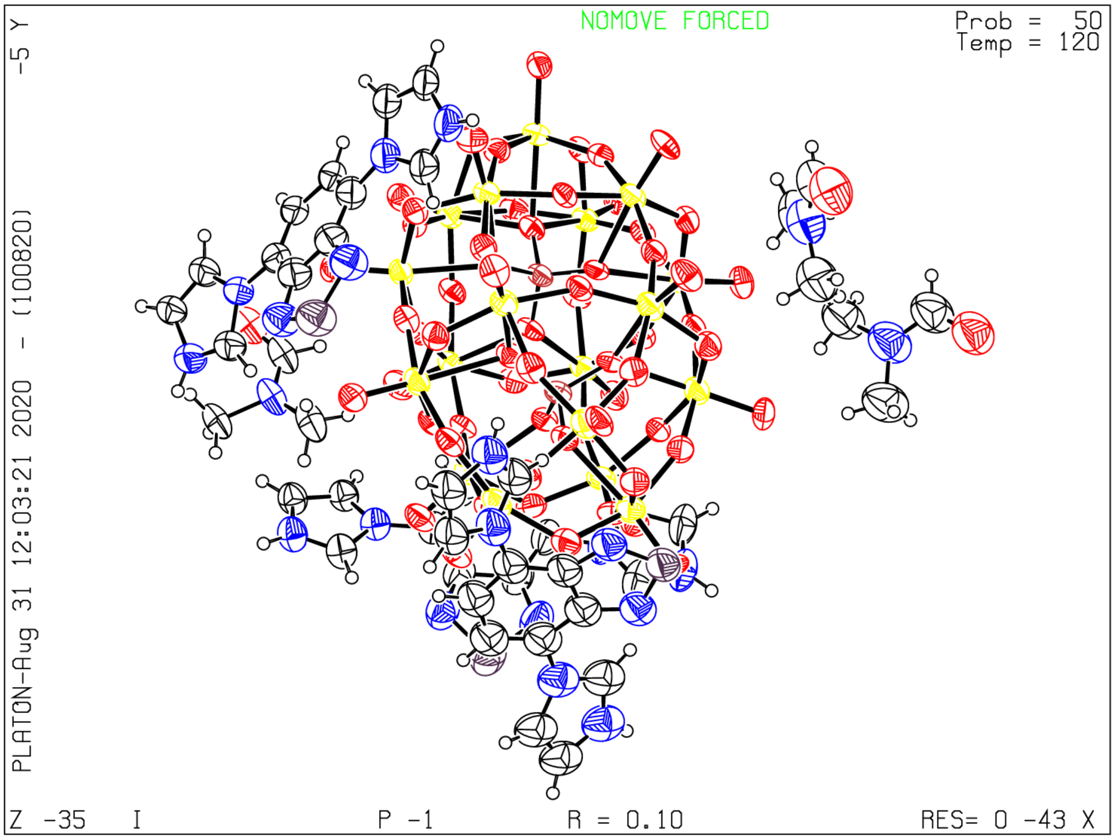

Supplement: Supplementary file 2 [file Data_Sheet_1.PDF]
